# Biomechanical modeling of musculoskeletal function related to the terrestrial locomotion of *Riojasuchus tenuisceps* (Archosauria: Ornithosuchidae)

**DOI:** 10.1002/ar.25528

**Published:** 2024-06-29

**Authors:** M. Belen von Baczko, Juned Zariwala, Sarah Elizabeth Ballentine, Julia B. Desojo, John R. Hutchinson

**Affiliations:** ^1^ Sección Paleontología de Vertebrados Museo Argentino de Ciencias Naturales Bernardino Rivadavia Ciudad Autónoma de Buenos Aires Argentina; ^2^ Consejo Nacional de Investigaciones Científicas y Técnicas (CONICET) Ciudad Autónoma de Buenos Aires Argentina; ^3^ Structure & Motion Laboratory, Department of Comparative Biomedical Sciences Royal Veterinary College Hatfield Hertfordshire UK; ^4^ School of Life and Environmental Sciences, College of Health and Sciences University of Lincoln, Brayford Pool Campus Lincoln UK; ^5^ División Paleontología Vertebrados Facultad de Ciencias Naturales y Museo La Plata Argentina

**Keywords:** bipedalism, locomotion, modeling, pseudosuchia, Triassic

## Abstract

*Riojasuchus tenuisceps* was a pseudosuchian archosaur from the Late Triassic period in Argentina. Like other ornithosuchids, it had unusual morphology such as a unique “crocodile‐reversed” ankle joint, a lesser trochanter as in dinosaurs and a few other archosaurs, robust vertebrae, and somewhat shortened, gracile forelimbs. Such traits have fuelled controversies about its locomotor function—were its limbs erect or “semi‐erect”? Was it quadrupedal or bipedal, or a mixture thereof? These controversies seem to persist because analyses have been qualitative (functional morphology) or correlative (morphometrics) rather than explicitly, quantitatively testing mechanistic hypotheses about locomotor function. Here, we develop a 3D whole‐body model of *R. tenuisceps* with the musculoskeletal apparatus of the hindlimbs represented in detail using a new muscle reconstruction. We use this model to quantify the body dimensions and hindlimb muscle leverages of this enigmatic taxon, and to estimate joint ranges of motion and qualitative joint functions. Our model supports prior arguments that *R. tenuisceps* used an erect posture, parasagittal gait and plantigrade pes. However, some of our inferences illuminate the rather contradictory nature of evidence from the musculoskeletal system of *R. tenuisceps*—different features support (or are ambiguous regarding) quadrupedalism or bipedalism. Deeper analyses of our biomechanical model could move toward a consensus regarding ornithosuchid locomotion. Answering these questions would not only help understand the palaeobiology and bizarre morphology of this clade, but also more broadly if (or how) locomotor abilities played a role in the survival versus extinction of various archosaur lineages during the end‐Triassic mass extinction event.

## 1 INTRODUCTION

**FIGURE 1:**
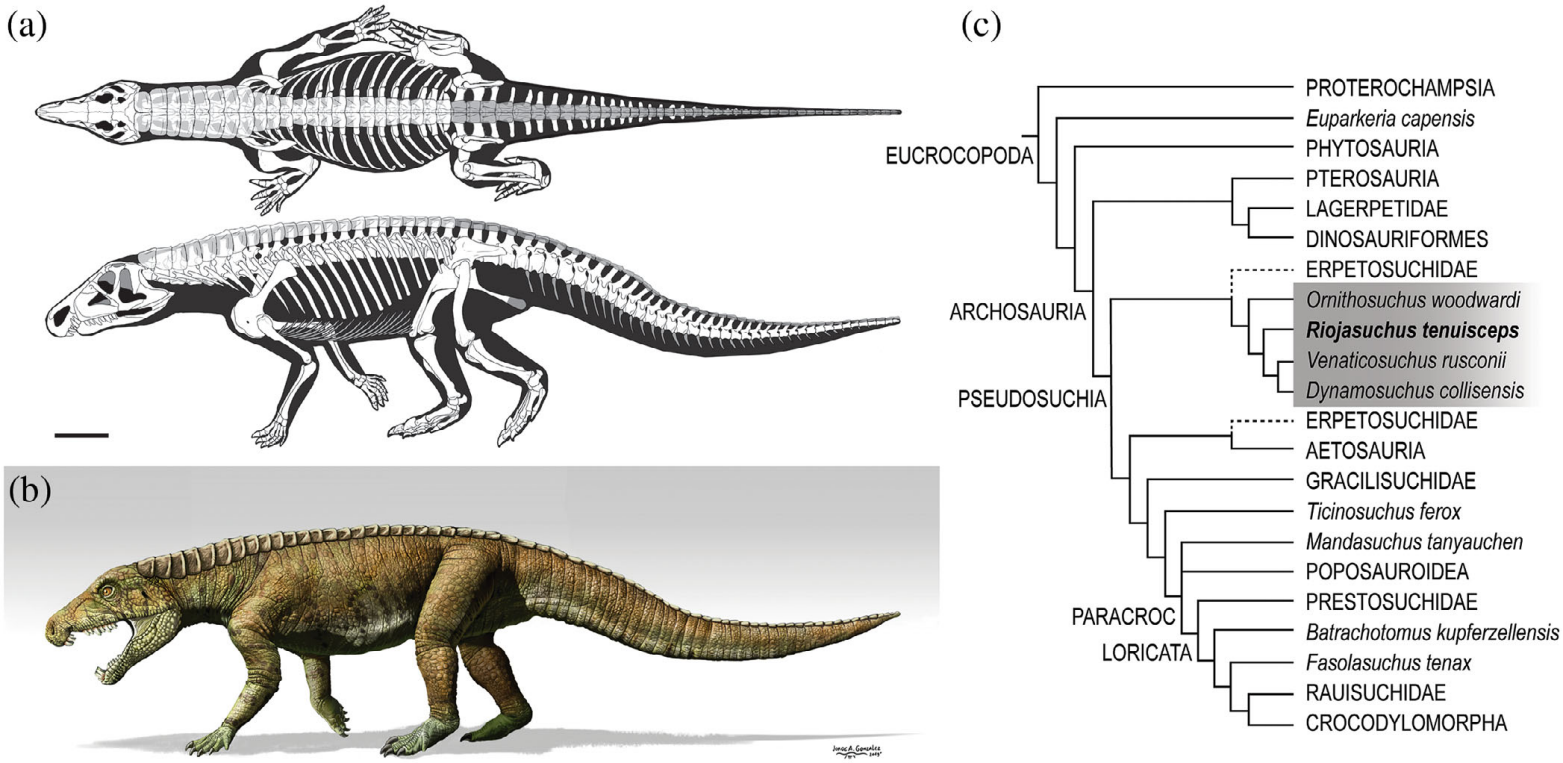
Skeletal reconstruction in dorsal and lateral view (a), life reconstruction (b) of *Riojasuchus tenuisceps*; artist: Jorge Gonzalez; and (c) phylogeny of Eucrocrocopoda including Archosauria, showing recently proposed phylogenetic relationships of *R. tenuisceps* with other key taxa including some discussed in the main text. Ornithosuchidae is highlighted in gray. “PARACROC”, Paracrocodylomorpha.

Ornithosuchidae is a clade of terrestrial, medium‐sized pseudosuchian (crocodile‐line or stem crocodylian) archosaurs known from the Upper Triassic deposits of South America and Europe, currently represented by four species: *Ornithosuchus woodwardi* (Lossiemouth Sandstone Formation, Scotland), *Riojasuchus tenuisceps* (Los Colorados Formation, Argentina), *Venaticosuchus rusconii* (Ischigualasto Formation, Argentina), and *Dynamosuchus collisensis* (Santa Maria Formation, Brazil) (Bonaparte, 1972; Müller et al., 2020; von Baczko & Ezcurra, 2013; Walker, 1964). In particular, *R. tenuisceps* (Figure 1a,b) is an outstanding representative of this group because of its excellent three‐dimensional preservation of several specimens, including two nearly complete skeletons.

*Riojasuchus* has some peculiar cranial and postcranial features including a triangular skull with a remarkably narrow snout, a downturned premaxilla, a large diastema between the premaxilla and maxilla, large caniniform teeth on the mandible that fit into the diastema when the jaws occlude, jaws shorter than the skull, palatine‐pterygoid fenestrae, large external nares and a deep antorbital fossa surrounding the fenestra (von Baczko & Desojo, 2016). Among the notable postcranial features of *Riojasuchus*, the cervical vertebrae are robust and have marked spine tables, the sacrum has three rather than the ancestral two vertebrae incorporated, the preserved caudal vertebrae have long transverse processes and neural spines, the forelimbs are ~25% shorter than the hindlimbs, the pelvis has an incipiently perforated acetabulum, a short preacetabular process of the ilium and an elongated pubis with a well‐developed pubic apron, the femur is sigmoid‐shaped and has a well‐developed lesser (anterior) trochanter (convergent with Dinosauriformes), and the ankle joint—as a unique trait of Ornithosuchidae—has a “crocodile‐reversed” condition in which the calcaneum has a condyle that fits into a cotyle of the astragalus (Parrish, 1986; Sereno, 1991; von Baczko et al., 2020; Walker, 1964). This array of features has led to some controversy about the phylogenetic relationships for ornithosuchids, at first considered as the origin of theropod dinosaurs (Walker, 1964), and later interpreted as early‐diverging bird‐line or pseudosuchian archosaurs (Benton & Clark, 1988; Gauthier, 1986; Nesbitt, 2011; Parrish, 1993; Sereno, 1991). Recently, Ornithosuchidae is considered nested within Pseudosuchia as a sister group to Erpetosuchidae (Ezcurra et al., 2017) or Erpetosuchidae + Aetosauria (Ezcurra et al., 2020). Within Ornithosuchidae, *R. tenuisceps* has been recovered as a later‐diverging species more closely related to *Venaticosuchus* and *Dynamosuchus* from South America than to *Ornithosuchus* from Scotland (Müller et al., 2020). Nonetheless, all studies since the 1980s (and more ambiguously, earlier studies treating the clade as ‘thecodonts’) have regarded Ornithosuchidae as embedded within Archosauria and the slightly broader clade Eucrocopoda (Figure 1c).

The palaeobiology of ornithosuchids is still a puzzling issue, with different proposals concerning their feeding behavior and their locomotor abilities. Originally proposed as either hunters or scavengers based on their anatomical features and probable traces (Benton, 1983; Walker, 1964), a more recent study has supported a scavenging habit based on the moment arms of the jaw musculature and bite force (von Baczko, 2018). However, an alternative hypothesis has been proposed as well, based on a three‐dimensional finite element analysis of the skull and lower jaw of *R. tenuisceps*, suggesting that it fed on small prey caught along river banks (zoophagous diet) helped by a possible wading habit (Taborda et al., 2023).

The terrestrial locomotor abilities of ornithosuchids have long been contentious. This controversy is important because they are an unusual clade of Triassic pseudosuchians that give insight into locomotor diversity in Archosauria, and thereby potentially also into longstanding broader controversies including the factors that may explain archosaurian survival versus extinction in the end‐Triassic mass extinction (for a recent review, see Cuff et al., 2022). Ornithosuchids have been interpreted by different studies as bipedal (Walker, 1964), quadrupedal with bipedal faculties (Bonaparte, 1972) because of the disparity in length between their forelimbs and hindlimbs (especially their shorter manual vs. longer pedal digits); or perhaps quadrupedal (Demuth, Wiseman, and Hutchinson, 2023; Grinham et al., 2019; Kubo & Kubo, 2012; Sennikov, 2024). Pintore et al.'s (2022) 3D geometric morphometrics analysis of archosauriform femora predicted that *Riojasuchus* was, depending on which of two specimens was used, either quadrupedal or bipedal (their tab. 2); but also showed that its femur was remarkably robust (also commented on by Sennikov, 2024). Bishop et al. (2020) also had similarly ambiguous results; in their case using statistical analysis of morphometric data from limb lengths and body segment mass properties estimated using a simple model. The ankle joint of ornithosuchids is also a critical feature to consider, because it suggests a plantigrade posture of the feet with a crurotarsal rotation of the ankle, as opposed to the digitigrade posture and “advanced mesotarsal” ankle seen for example in dinosaurs. There even remains some confusion in the literature over whether ornithosuchids had more erect (adducted) or more sprawling (abducted) limb postures (e.g., Cruickshank & Benton, 1985; Parrish, 1986; Sennikov, 2024; Sereno, 1991; Sullivan, 2015; Walker, 1964); and while it is generally thought that their pes (and perhaps manus) adopted plantigrade poses, that issue has not been deeply investigated (but see Parrish, 1986; Sennikov, 2024).

A new perspective on the biomechanical capacities of terrestrial locomotion is needed to test many locomotor hypotheses for *Riojasuchus*, and by logical extension for ornithosuchids in general. Here, in order to achieve our aims, we: (1) construct a composite three‐dimensional (3D) musculoskeletal model of the hindlimbs of *Riojasuchus*, and a whole‐body model of its mass properties (masses and centres of mass [COMs]), testing if the body dimensions would facilitate bipedalism; (2) use the model's basic joint morphology to infer what segment orientations (e.g., erect posture; plantigrade pes) might have been achieved and crude estimates of joint ranges of motion (ROMs); (3) estimate its hindlimb muscle moment arms (MMAs); and (4) compare results from aims #1 to #3 with those from other archosauriform taxa to explore different musculoskeletal specializations and evolutionary changes, facilitating future assessment of their potential relevance to the extinction and survival of different archosaur clades across the Triassic–Jurassic boundary.

## 2 MATERIALS AND METHODS

### 2.1 Specimen choice and scanning

**FIGURE 2:**
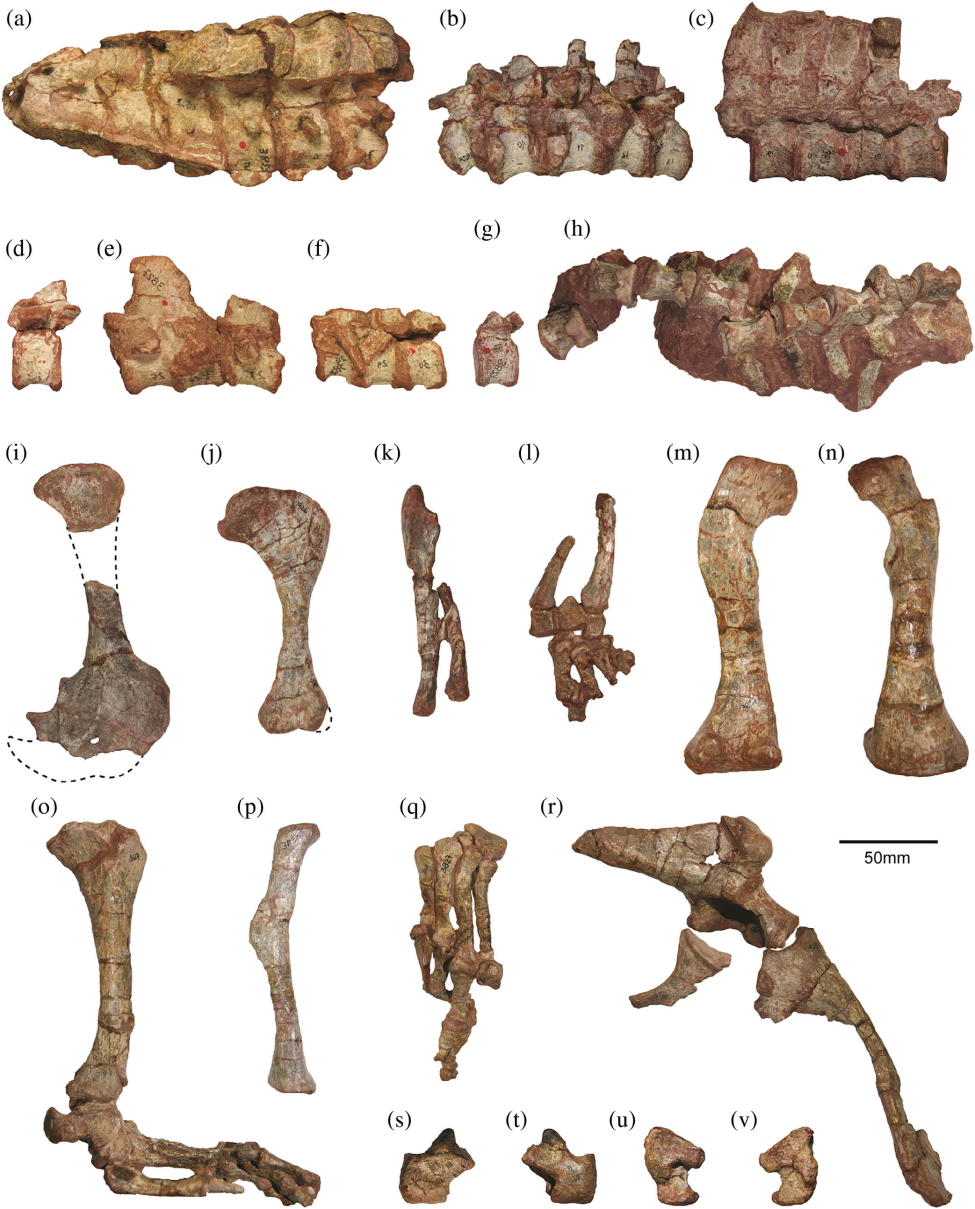
Postcranial elements of *Riojasuchus tenuisceps*. Articulated cervical (a), cervicodorsal (b), dorsal (c), 24th dorsal (d), sacral (e), proximal caudal (f), and middle caudal vertebrae (g), middle caudal vertebrae (h), left scapula and coracoid (i), right humerus (j), left ulna and radius (k), articulated left manus, ulna and radius (l), left femur (m, n), articulated right tibia, tarsus and pes (o), left fibula (p), articulated left pes (q), left pelvic girdle (r), left astragalus (s, t), left calcaneum (u, v). Pictures of vertebral series a–g, ilium and pubis are mirrored.

The postcranial elements of specimens PVL (Colección Paleontología de Vertebrados Lillo, Facultad de Ciencias Naturales e Instituto Miguel Lillo, Universidad Nacional de Tucumán, Tucumán, Argentina) 3826, 3827 and 3828 were scanned at Diagnósticos Gamma (San Miguel de Tucumán, Tucumán, Argentina), in a Philips Gemini TF 16‐channel axial CT scanner at slice thickness of 0.8 mm and 0.4 mm of overlap, voxel size of 0.39 × 0.39 × 0.4 mm, penetration power of 120.0 kV and 279 mA. The obtained images were thresholded and segmented using the open‐source software 3D Slicer v4.8.1 (Fedorov et al., 2012). The isolated atlas of PVL 3826 was digitized through photogrammetry using the software Agisoft Photoscan v1.0.3 (https://www.agisoft.com). All 3D models were exported as .OBJ polygonal mesh files. Table 1 shows what bones ultimately were used in the final model, and Figure 2 summarizes key postcranial elements. While we digitally smoothed most bone meshes, we did not correct for taphonomic deformations. These were deemed modest for the hindlimb (see Bonaparte, 1972; von Baczko et al., 2020); an exception is the distal tibial shaft which is deformed, but its morphology would have minimal impact on our analyses. The humerus's joint morphology was improved by merging the scaled mesh of PVL 3827 (proximal and distal ends) with the whole humerus of PVL 3826, but the humerus was not used here for other analyses. All three main specimens used were discovered together in the field, and are of similar size (von Baczko et al., 2020), so allometric concerns are not a problem for our model.

**TABLE 1:** Skeletal elements, specimens, and numbers of CT scan X‐ray slices used in the model.

Skeletal element	Specimen	CT slice count
Atlas	PVL 3826	N/A
Cervical vertebrae (1–7)	PVL 3827	594
Cervical vertebra 8	PVL 3827	250
Cervicodorsal vertebrae (9–13)	PVL 3827	374
Dorsal vertebrae (19–22)	PVL 3827	384
Dorsal vertebra 24	PVL 3827	256
Sacral vertebrae (1–3)	PVL 3827	356
Proximal caudal vertebrae (1–3)	PVL 3827	267
Mid‐caudal vertebrae (~4–12^a^)	PVL 3828	607
Partial scapula and coracoid (right)	PVL 3827	299
Scapular blade (left)	PVL 3826	171
Coracoid (left)	PVL 3828	275
Humerus (right)	PVL 3826 and 3827	406
Ulna (right)	PVL 3828	374
Radius (right, left) (mid‐shaft missing)	PVL 3827 and 3828	169 and 374
Articulated manus (left)	PVL 3827	358
Ilium (left)	PVL 3828	382
Ischium (left)	PVL 3828	200
Pubis (left)	PVL 3827	511
Femur (left)	PVL 3828	540
Tibia + fibula + articulated pes (right)	PVL 3827	615
Articulated pes (left)	PVL 3827	437

^a^ The actual positions of these caudal vertebrae might have been closer to caudal numbers 7–15, but we implemented them as continuous with the proximalmost caudals.

### 2.2 Skeletal model construction

We followed the methods for skeletal model (‘digital marionette’) construction detailed by Bishop, Cuff, and Hutchinson (2021) and used in other papers by Bishop, Falisse, et al. (2021), Bishop et al. (2021), Demuth et al. (2020), Demuth, Wiseman, and Hutchinson (2023) and Wiseman et al. (2021). Polygonal meshes (.OBJ format) were decimated to <50,000 polygons in MeshLab v2021 software (Cignoni et al., 2008; https://www.meshlab.net/). The bones were then posed into an initial “reference pose” (also known as “neutral posture”), with vertebrae manually articulated in order to maximize intercentral and zygapophyseal articulations, and the limbs posed vertically except for a plantigrade pes parallel to the “substrate”. The manus was maintained vertical (“digitigrade”) due to uncertainty about its posture (see Section 3). PVL 3827 was used as the focal specimen and other bones of specimens used (Table 1) were scaled to match its relative dimensions (vs. overlapping elements), or missing vertebrae (e.g., presacrals 14–18) scaled to match relative dimensions of preserved vertebrae based on trends for length and width across that region of the vertebral column. A missing part of the scapular shaft was scaled from a mesh file of the scapula of *Euparkeria capensis* (Demuth, Wiseman, & Hutchinson, 2023), because the morphological differences between its shape and that of pseudosuchians was deemed minor. The distal ischia were manually reconstructed as a mesh following the reconstruction (here, Figure 1a) in von Baczko et al. (2020).

The articulated skeleton was then given anatomical coordinate systems (ACSs) following Bishop, Cuff, and Hutchinson (2021) and Gatesy et al. (2022) and references therein. Briefly, articular surfaces (centra) were digitally isolated in MeshLab for the intervertebral joints at the 7th–8th, 20th–21st, and sacrocaudal articulations; and for the ends of the sacrum (used to produce a craniocaudal axis of the model). Similarly, the articular surfaces of the (forelimb) glenoid and proximal and distal humerus, radius and ulna and (hindlimb) acetabulum, femur, tibia and fibula, proximal tarsals, and metatarsal III were isolated. Because of taphonomic distortion and small size, we manually adjusted the wrist, metacarpophalangeal, and metatarsophalangeal ACSs. We fit geometric primitives to those surfaces as follows: cylinders for the vertebrae and distal ends of all limb bones (except as follows); spheres for the glenoid, proximal humerus, proximal and distal radius and ulna; an ellipsoid for the proximal femur; and planes for the proximal ends of limb bones (except radius and ulna). The bone and geometric primitives files were then loaded into Rhinoceros v7.0 (McNeel and Associates, Barcelona, Spain) software for further model construction.

We used the MATLAB (v2023; MathWorks, Inc., Natick, MA, USA) scripts from Bishop, Cuff, and Hutchinson (2021) to automatically generate right‐handed ACSs (*x*, *y*, and *z* vectors) from those geometric primitives. Next, we matched these ACSs to the model in Rhinoceros, in order to use the ACSs to define joint coordinate systems (JCSs); again following Bishop, Cuff, and Hutchinson (2021) and Gatesy et al. (2022) and references therein. Geometry was then re‐posed as necessary to bring the reference pose values of joints as close to 0° as possible (i.e., straight proximal limb segments in the sagittal plane) and minimize bone overlap as necessary. Joints were only allowed rotations; no translations (see Manafzadeh & Gatesy, 2021, 2022, and references therein for limitations), in an *x*, *y*, and *z* axis rotation order in later modeling. As in prior work, we defined (positive/negative angles) extension/flexion of limb joints as occurring about the JCS's *z*‐axis; abduction/adduction as about the *y*‐axis; and external/internal long‐axis rotation as about the *x*‐axis. Intervertebral JCSs essentially followed Bishop, Falisse, et al. (2021) but are not a focus here; they were left at the reference pose. Limb joints were given a single degree of freedom (DOF) for all but the hip and shoulder joints, which had three DOF. At the final stage, limb joints were translated as necessary to add space for articular cartilage (Holliday et al., 2010), following our prior standards (Bishop, Cuff, & Hutchinson, 2021; Hutchinson et al., 2005). As the hip and ankle joints were very tightly articulating, no extra space was added to these.

### 2.3 Hindlimb joint morphology, joint ROMs and qualitative segment orientations

We used the reconstructed morphology of the hindlimbs to qualitatively assess likely segment orientations and articulations (e.g., in a representative standing pose), with comparisons to the literature (see Section 3). Next, we followed basic practices for estimating joint ROMs one DOF at a time, which makes many assumptions such as the absence of joint translation and interactions of DOFs across ‘joint mobility’ hypervolumes. The relatively crude approach we used (followed by other studies such as Bishop, Cuff, & Hutchinson, 2021; Otero et al., 2017; Pierce et al., 2012) simply manually positioned joints (here, in OpenSim; see Section 2.5) and relatively subjectively inspected for where excessive disarticulation or bone collision happened, using those positions as ROM limits in the final model. The resulting estimates form a sensible starting point that more rigorous 3D methods can build on using our models in the future.

### 2.4 Reconstructing body dimensions and mass properties

**FIGURE 3:**
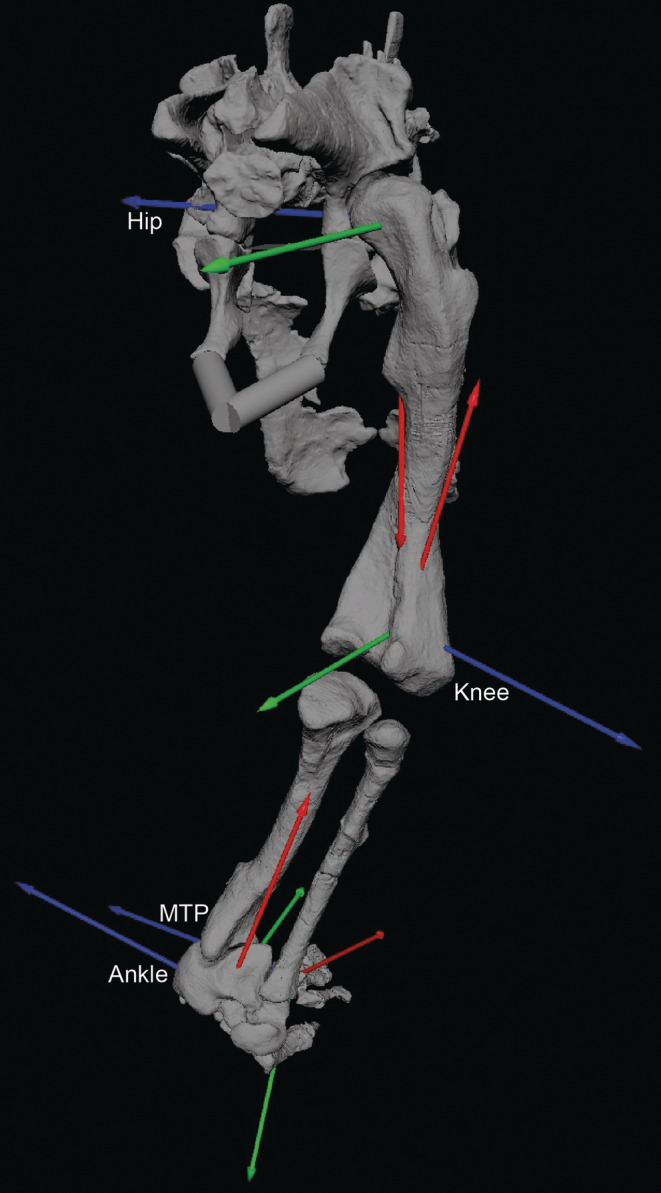
Right hindlimb JCSs for our model of *Riojasuchus*, in oblique caudal view. These follow standard conventions cited in Section 2; for example, red, green, and blue axes = long‐axis rotation, adduction/abduction, and flexion/extension. Joints (hip, knee, ankle, and MTP) are labeled next to their flexion/extension axes. Arrows point toward positive values of angles. Not to scale.

Because the skeleton is only a fraction of the size of the actual organism, and we sought to do biomechanical analyses of the whole *Riojasuchus* organism, we used our team's prior methods (Allen et al., 2009, 2013; Hutchinson et al., 2007 and other studies), formalized by Bishop, Cuff, and Hutchinson (2021), to estimate the body shape of *Riojasuchus* and use those dimensions to compute segmental mass properties. We fit multiple octagonal polygonal hoops serially along the longitudinal axis for each major body segment craniocaudally (axial segments) or proximodistally (limb segments) (head and neck, front and back halves of torso), “body” (pelvis‐sacrum), proximal and distal tail, [forelimb:] upper and forearm, manus, manual digits, [hindlimb:] thigh, crus/shank, pes and pedal digits. As there are only 13 (proximalmost) caudal vertebrae known for *Riojasuchus*, we used the scaled scientific reconstruction (here, Figure 1) from von Baczko et al. (2020); and three isolated distal caudals of serially decreasing height and increasing length; to estimate the dimensions of the missing distal tail. We added internal spaces to represent air cavities in the cranium (pharynx, sinuses), neck (trachea), and thorax (lungs) using the same octagon‐based polygonal hoop‐building approach. The initial hoops closely followed skeletal outlines except around the proximal ends of limb segments or where skeletal material was missing (e.g., ribs, gastralia, and caudal chevrons), so this “shrink‐wrapping” method is analogous to convex hulls used in other studies (Brassey et al., 2016; Clauss et al., 2017; Macaulay et al., 2023; Maher et al., 2022; Sellers et al., 2012). However, as per our prior studies we then expanded these hoops to represent missing soft tissues (because living whole organisms are not shrink‐wrapped), producing a mean 3D shape for each segment as we lofted the hoops together to connect adjacent hoops. All segments were mirrored right‐to‐left as necessary to produce a mediolaterally symmetrical model. That Rhinoceros model's geometry (bones and final lofted body segments) was then exported as .OBJ meshes, resaved as triangular meshes in Meshlab, and the coordinates of the ACSs were recorded. The latter were used in Autodesk Maya v2023 software (San Francisco, CA) to construct the JCSs (Brainerd et al., 2010; Gatesy et al., 2022) using the XrommTools plugin (https://bitbucket.org/xromm/xromm_mayatools/src/master/), exporting JCSs as .OBJ files. Figure 3 shows the final hindlimb JCSs. As the focus of our study is on hindlimb function, we do not detail forelimb or other postcranial joints here.

We do, however, address if the new whole‐body mass properties (and lengths of the forelimbs and hindlimbs) output for our model alter the results of the analysis by Bishop et al. (2020), who used a 3D model of *Riojasuchus* from Henderson and Snively (2004) in a statistical, morphometric analysis of models of 80 archosauriforms using a training dataset for linear discriminant analysis (LDA). For details, see Bishop et al. (2020); but briefly, we re‐ran their LDA analysis with our new input data for body mass and centre of mass (COM; made dimensionless by dividing by gleno‐acetabular distance); and forelimb and hindlimb lengths; using the MASS package for R (v. 7.3‐50; Venables & Ripley, 2002). The LDA involved 22 different training datasets, each of which was used to predict locomotor postures for up to 66 taxa and compare the prediction's success rate to pre‐determined “known” postures for these.

### 2.5 OpenSim musculoskeletal model construction

**FIGURE 4:**
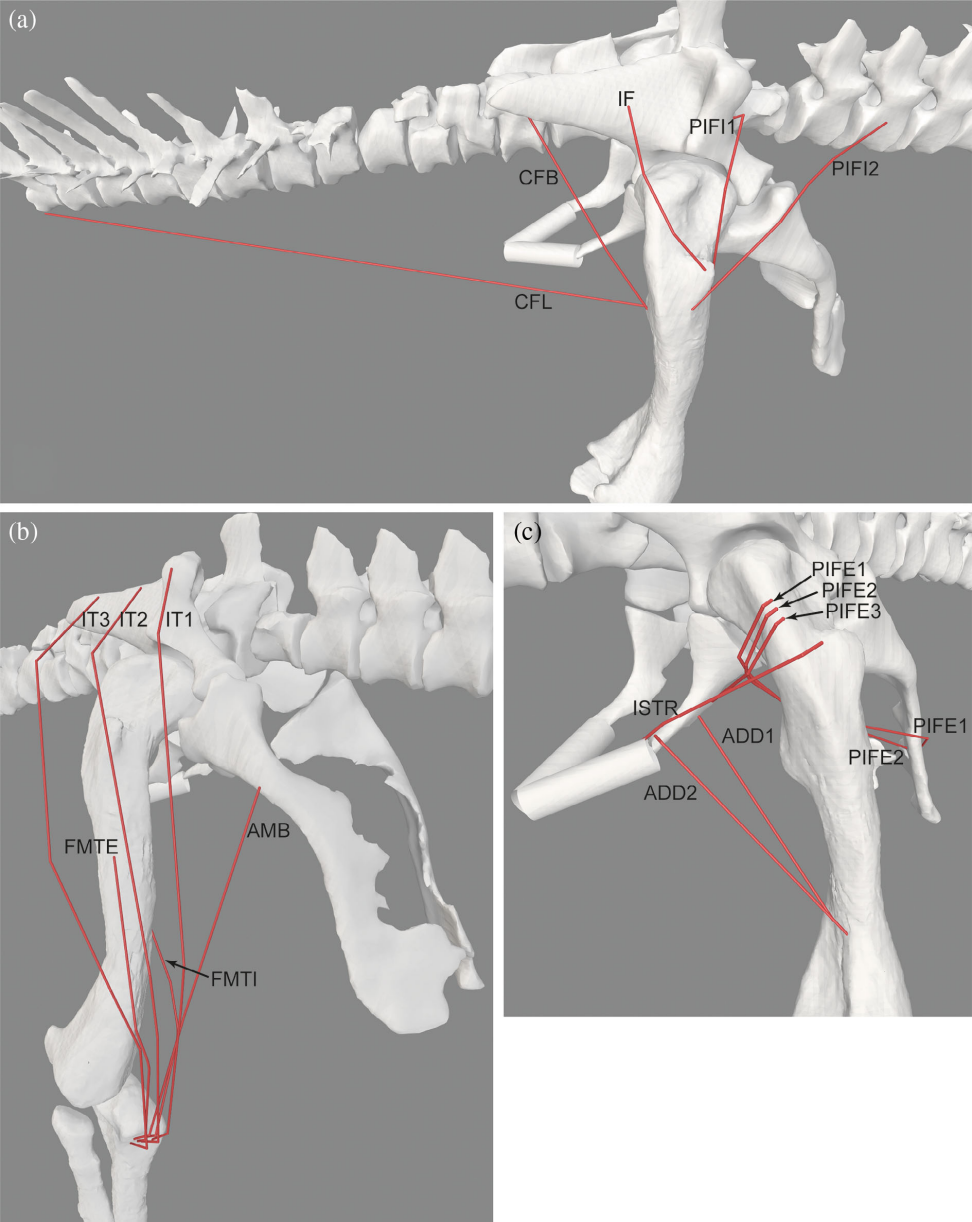
Right hindlimb musculature reconstructed for *Riojasuchus*. (a) Caudodorsal/lateral view of major deep hip flexors and extensors. (b) Craniolateral view of “triceps femoris” knee extensors. (c) Caudolateral view of more deep hip flexors and extensors. See Table 2 for acronyms. Not to scale.

**FIGURE 5:**
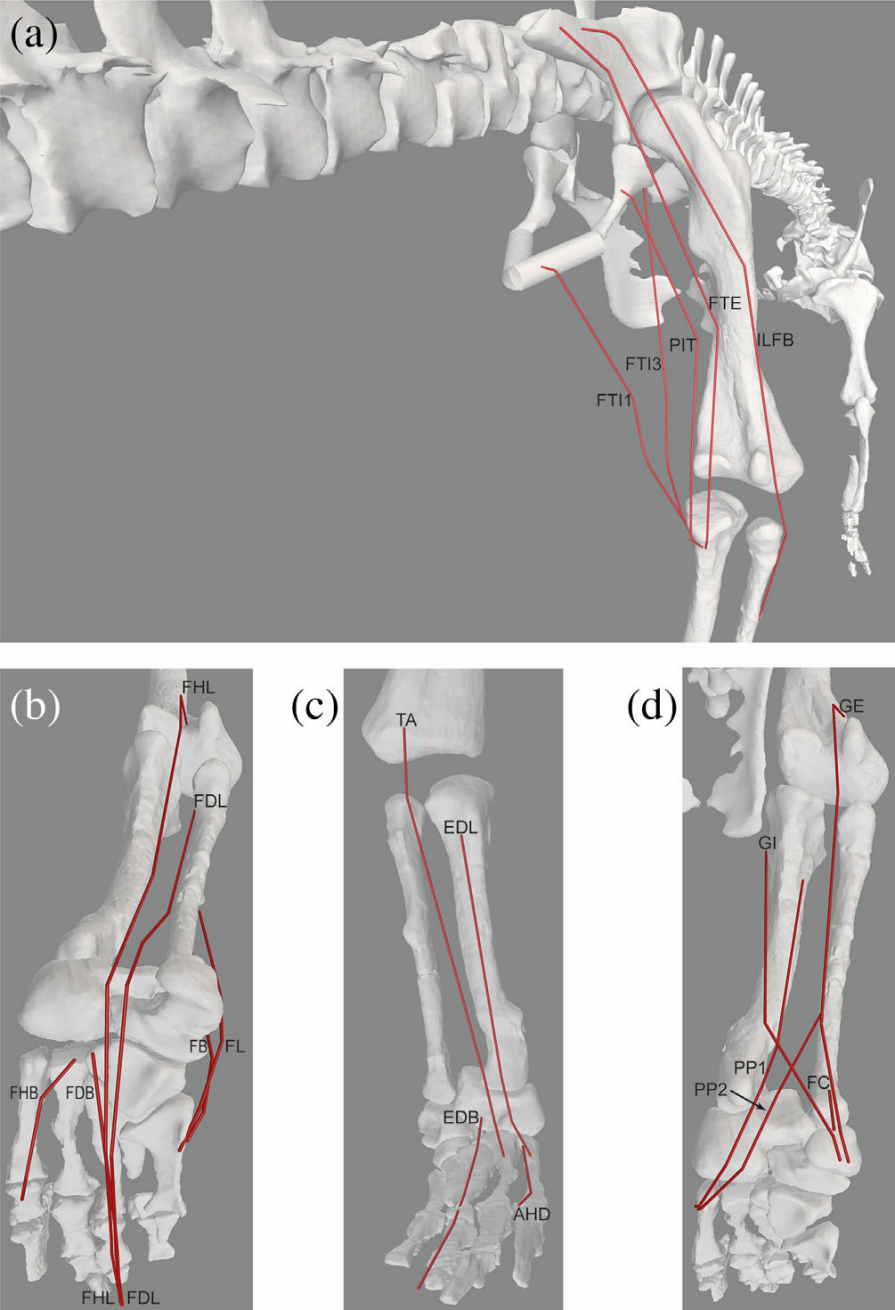
Right hindlimb musculature reconstructed for *Riojasuchus*. (a) Caudal view of hip extensors and knee flexors. (b) Caudoventral view of ankle extensors and digital plantarflexors. (c) Cranial view of ankle flexors and digital dorsiflexors. (d) Caudoventral view of ankle extensors. See Table 2 for acronyms. Not to scale.

The geometry exported from Rhinoceros and Maya software then was processed with custom MATLAB code to produce an OpenSim v4.5 (Seth et al., 2018; https://simtk.org/projects/opensim) .osim format model and geometry files were transformed to match OpenSim's format for constructing dynamic chains in a 3D coordinate system. Finally, for use in biomechanical simulation, we reconstructed the hindlimb 3D muscle geometry of *Riojasuchus* in OpenSim. Reconstruction of archosaurian hindlimb muscle paths is based on a strong foundation of data on osteological correlates of muscle attachments detailed by Hutchinson (2001a, 2001b, 2002), also Bishop, Cuff, and Hutchinson (2021), using the method of the Extant Phylogenetic Bracket (EPB; Witmer, 1995). We analyzed these data via maximum parsimony character tracing in Mesquite v3.81 software (Maddison & Maddison, 2023; http://www.mesquiteproject.org) to produce predicted character states in *Riojasuchus*; detailed in Appendix S1 and listed in Table 2; including muscle names and acronyms used here as well as ‘levels of inference’ sensu Witmer (1995). We positioned these attachments in OpenSim and added ‘via points’ and ‘wrapping surfaces’ to constrain the 3D muscle paths, following protocols from Hutchinson et al. (2005, 2015), Allen et al. (2021) and Bishop, Cuff, and Hutchinson (2021), among others. Positioning of the attachments involved inspecting the original bones as well as the scanned representations for osteological correlates. The small intrinsic muscles of the pes were not reconstructed. The resulting musculoskeletal model was refined to ensure that muscles did not cut through bones or each other excessively throughout the approximate joint ROMs, and is shown in Figures 4 and 5.

**TABLE 2:** *Riojasuchus* hindlimb muscle origins and insertions reconstructed.

Muscle	Origin	Insertion
M. iliotibialis 1 [IT1]	Craniodorsal iliac rim (roughening) [I]	Cranial tip of cnemial crest of tibia [I]
M. iliotibialis 2 [IT2]	Mid‐dorsal iliac rim (roughening) [I]	Cranial tip of cnemial crest of tibia [I]
M. iliotibialis 3 [IT3]	Caudodorsal iliac rim (roughening) [I]	Cranial tip of cnemial crest of tibia [I]
M. femorotibialis externus [FMTE]	Lateral femoral shaft, between intermuscular lines [I]	Cnemial crest of tibia [I]
M. femorotibialis internus [FMTI]	Medial femoral shaft, between intermuscular lines and other muscle scars [I]	Cnemial crest of tibia [I]
M. ambiens [AMB]	Pubic tubercle of proximal pubis [I]	Cnemial crest of tibia [I]; secondary tendon to digital flexor origin [I']
M. iliofibularis [ILFB]	Lateral surface of postacetabular ilium, between IF and FTE [I]	Iliofibular tubercle on lateral proximal fibular shaft [I]
M. iliofemoralis [IF]	Lateral surface of ilium above acetabulum [II]	Lesser trochanter of proximal femur [II]
M. pubo‐ischio‐femoralis internus 1 [PIFI1]	Craniomedial side of preacetabular ilium [II]	Craniomedial proximal femoral shaft, lateral to fourth trochanter [II]
M. pubo‐ischio‐femoralis internus 2 [PIFI2]	“Lumbar” (dorsal) vertebrae close to preacetabular ilium; lateral central surfaces [II]	Craniolateral proximal femur, near lesser trochanter [I']
M. pubo‐ischio‐tibialis (PIT)	PIT (one head) on craniolateral proximal ischial apron, craniad to other ischial muscles [II]	Medial proximal tibia [I']
M. flexor tibialis internus 1 (FTI1)	Lateral surface of distal ischial shaft (tubercle/scar) [II']	Medial proximal tibia [I']
M. flexor tibialis internus 3 (FTI3)	Proximal ischial tuberosity (scar) [II]	Caudal proximal tibia [I']
M. flexor tibialis externus (FTE)	Lateral surface of caudoventral corner of postacetabular ilium [I']	Caudal proximal tibia [I']
M. puboischiofemoralis externus 1 (PIFE1)	Cranial surface of pubic apron [I]	Greater trochanter of femur [I]
M. puboischiofemoralis externus 2 (PIFE2)	Caudal surface of pubic apron [I]	Greater trochanter of femur [I]
M. puboischiofemoralis externus 3 (PIFE3)	Lateral surface of ischial apron, caudal to ADD1 [I]	Greater trochanter of femur [I]
M. ischiotrochantericus (ISTR)	Medial surface of ischial apron [I]	Lateral side of proximal‐most femur, near PIFE1–3 [I]
M. caudofemoralis brevis (CFB)	“Brevis” fossa of ilium, and proximal caudal vertebrae [I]	Caudolateral side of proximal fourth trochanter [I]
M. caudofemoralis longus (CFL)	Lateral surfaces of haemal arches/chevrons and transverse processes of proximal caudal vertebrae [I]	Fourth trochanter of femur; medial pit [I]
M. adductor femoris 1 (ADD1)	Craniolateral surface of ischial apron and shaft [I′]	Caudomedial distal femoral shaft [I']
M. adductor femoris 2 (ADD2)	Caudolateral surface of dorsal ischial shaft, from scarred groove [I]	Caudolateral distal femoral shaft near caudal intermuscular line [I']
M. gastrocnemius internus (GI)	Medial side of cnemial crest of proximal tibia [I′]	Plantar aponeurosis to metatarsal V, process on distal tarsal 4, and calcaneal tuber, then to digits 2–4 with FDB [II']
M. gastrocnemius externus (GE)	Proximal to lateral femoral condyle [I′]	Plantar aponeurosis to metatarsal V and calcaneum, then to digit 5 [II']
M. extensor digitorum longus (EDL)	Lateral side of the cnemial crest; distal to TA origin; and the cranial tibial shaft [II]	Craniomedial surface of proximal metatarsal I [II]
M. extensor digitorum brevis (EDB)	Cranial surfaces of proximal tarsals [II′]	Dorsal surfaces of distal phalanges [II]
M. tibialis anterior (TA)	Craniolateral side of the distal femur, and lateral cnemial crest [II]	Craniomedial sides of proximal metatarsals II–IV [II′]
M. flexor digitorum longus (FDL)	Proximomedial fibula's shaft [I′]	Flexor tubercles of pedal unguals II–V [I]
M. flexor hallucis longus (FHL)	Caudolateral distal femur near GE origin, lateral cnemial crest of the tibia, fossa flexoria, and proximal fibula [II′]	Flexor tubercles of pedal unguals I–IV [I]
M. flexor digitorum brevis (FDB)	Plantar aponeurosis [II′]	Flexor tubercles of pedal unguals I–V [II]
M. flexor hallucis brevis (FHB)	Distal tarsals and plantar aponeurosis [II]	Caudal side of proximal digit 1, 1st phalanx; metatarsal I [II′]
M. fibularis longus (FL)	Lateral shaft of fibula, distal to ILFB insertion [I′]	Lateral side of metatarsal V; distal to FB; and calcaneal tuber [II]
M. fibularis brevis (FB)	Distalmost craniolateral shaft of fibula, distal to FL origin [I′]	Caudolateral side of metatarsal V (and IV); proximal to FL [II]
M. interosseous cruris/proximal pronator profundus (PP1)	Caudolateral proximal tibial shaft [II′]	Caudolateral side of metatarsal I and the process of distal tarsal IV [II]
M. pronator profundus (PP2)	Caudomedial fibular shaft [II′]	Caudolateral side of metatarsal I and the process of distal tarsal IV [II]
M. fibulocalcaneus (FC)	Caudal fibular surface, distal third [II′]	Dorsal surface of calcaneal tuber [II]
M. abductor hallucis dorsalis (AHD)	Craniolateral side of distal fibula [II′]	Proximodorsal surface of metatarsal I, near EDL insertion [II]

*Note*: Names [and acronyms] follow those used for Crocodylia (Hattori & Tsuihiji, 2021; Hutchinson, 2002; Pereyra et al., 2023; Romer, 1923a). Origins and insertions include levels of inference (Witmer, 1995) in [ ]: I, unequivocal; II, equivocal; with ′ denoting absence of a clear osteological correlate. Only muscles included in the musculoskeletal model are listed, often with simplified attachments because each muscle only had a single line of action (no branching origins or insertions).

### 2.6 Muscle moment arms

OpenSim can automatically output valuable data on muscle–tendon unit moment arms (MMAs) (‘leverages’) about any DOFs in a musculoskeletal model. These outputs can give a rough guide as to the actions; that is, from a simple static perspective, tendencies to cause/prevent motion and thereby achieve certain functions; of those muscles (Hutchinson et al., 2005, 2015) and in a comparative context can reveal the evolution of muscle function in Archosauria (Allen et al., 2021; Bates et al., 2015; Bates & Schachner, 2012; Cuff et al., 2022; Otero et al., 2017). We calculated the MMAs of all hindlimb muscles reconstructed versus the main joint DOFs in order to provide this guide about possible muscle actions, and then placed our findings into a comparative context in Section 3.

## 3 RESULTS AND DISCUSSION

Here we address our major aims, including how our model's mass properties relate to estimations of body mass and bipedal capabilities, then how joint morphology and joint ROMs (with a brief consideration of ichnological evidence and forelimb function) relate to hindlimb orientation and limb function, and finally our reconstructions of hindlimb myology and MMAs and how these compare with those for other archosaurs; including their evolutionary implications.

### 3.1 Whole body model, centre of mass, and bipedalism

**FIGURE 6:**
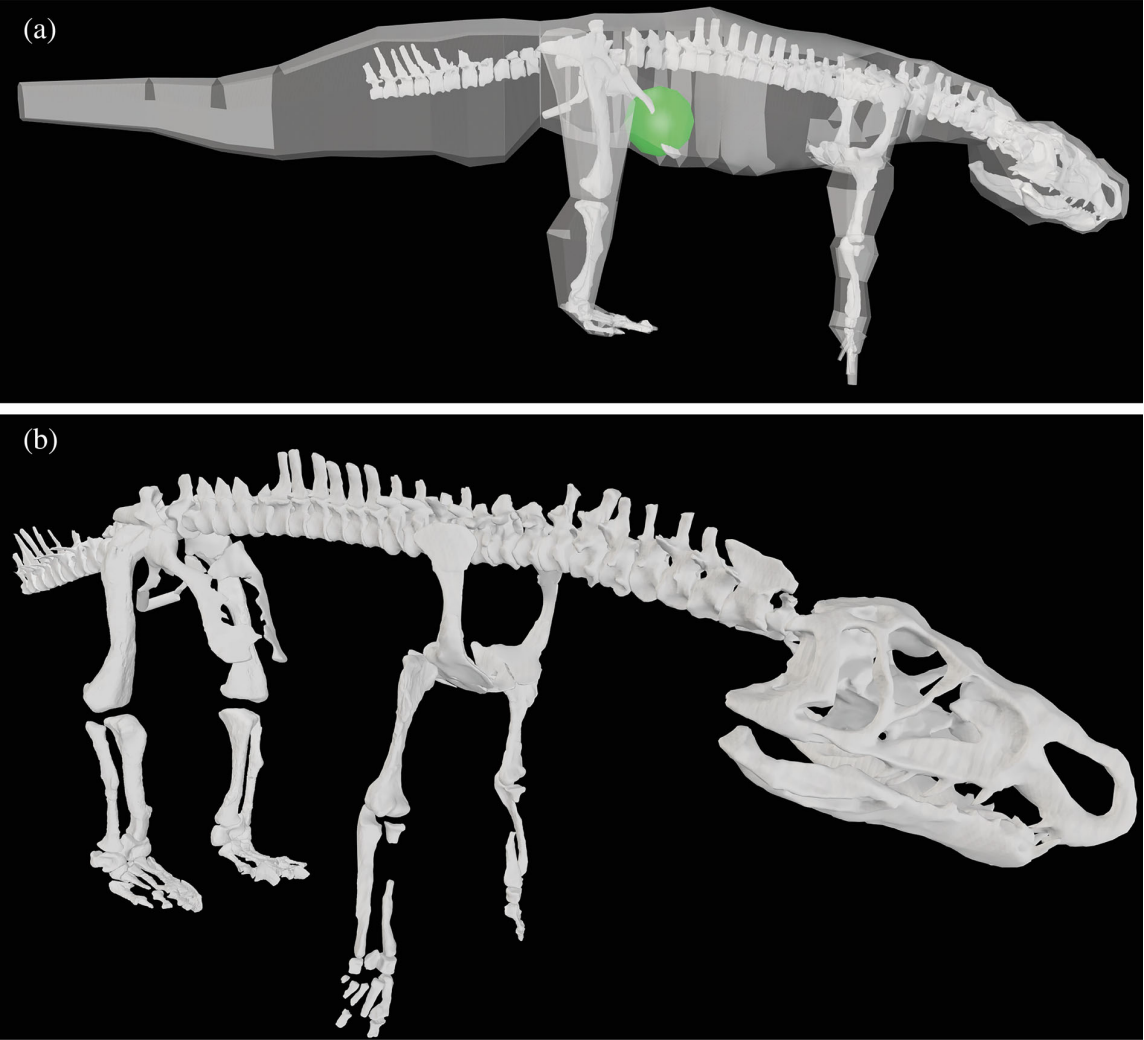
Whole‐body model of *Riojasuchus*. (a) Model (in right lateral view) with transparent objects representing segment shapes, used to calculate inertial properties; and whole‐body COM (green sphere). (b) Model (in oblique right lateral view) showing bones as per Table 1. Forelimbs and hindlimbs have been abducted by 15° from the reference pose at 0°. Not to scale.

Figure 6 shows the final whole‐body model and its centre of mass (COM). We obtained a total body mass of 24.56 kg for our model of *Riojasuchus*. This is very similar to an estimate of 28.33 kg (25.6% prediction error) from humeral and femoral minimal diaphyseal circumferences (~39 and 66 mm) using eq. (1) of Campione and Evans (2012), but less than the 41.6 kg estimate produced using the method of Hurlburt et al. (2003) based on skull length. It is considerably more than the estimated 13.55 kg from Henderson and Snively (2004) used in Bishop et al. (2020), and the COM position likewise was more craniad in that study (0.1364 m from hips vs. 0.0986 m here; see below); whereas the torso was longer (gleno‐acetabular distance = 0.400 m vs. 0.355 m in our model; and hindlimb and forelimb lengths = 0.420 m and 0.222 m vs. 0.320 m and 0.222 m in our model). It is unclear where these differences arose from. As Bishop et al.'s (2020) analyses used those different estimates and found that *Riojasuchus* possibly was quadrupedal (“misclassified as bipedal, 12 out of 22 times” in a training dataset for linear discriminant analysis [LDA]); also suggesting that this result was because of its quite caudal COM position; we re‐ran their analysis with our revised values. Somewhat surprisingly, we did not obtain qualitatively different results: the LDA predicted *Riojasuchus* as bipedal 12/22 times again, albeit with overall greater probabilities for bipedalism. Thus this method remains inconclusive for *Riojasuchus*.

We also used our whole‐body model to test how suited its COM was for bipedal support. To incorporate a slightly more biologically realistic pose, we adjusted the initial limb pose of the model to −10° flexion of the hip, knee, ankle, and elbow; and 10° flexion of the shoulder and wrist. The resulting whole body's COM was 0.0986 m craniad (and 0.0594 m ventrad) to the acetabula, compared with a femur length ~0.179 m; that is, about 50% of femur length craniad. Based on these measurements and the logic of Otero et al. (2019), *Riojasuchus* might have been able to sustain bipedalism in the default pose used, because it might have been able to place its COM over its feet (which had a length >0.11 m craniad to the acetabula in that pose), or even behind its knees if its limbs were more flexed than in our simple scenario (although evidence seems to point toward relatively vertically‐oriented limbs in *Riojasuchus*). However, our very basic, quasi‐biomechanical approach involves questionable assumptions such as requiring static equilibrium (necessary for bipedal standing, but not for facultative bipedalism; e.g., Demuth, Wiseman, & Hutchinson, 2023) and perhaps that a biped must have its COM behind its knee joint (see Bishop, Cuff, & Hutchinson, 2021, for commentary).

### 3.2 Joint morphology, ranges of motion, and limb poses

**FIGURE 7:**
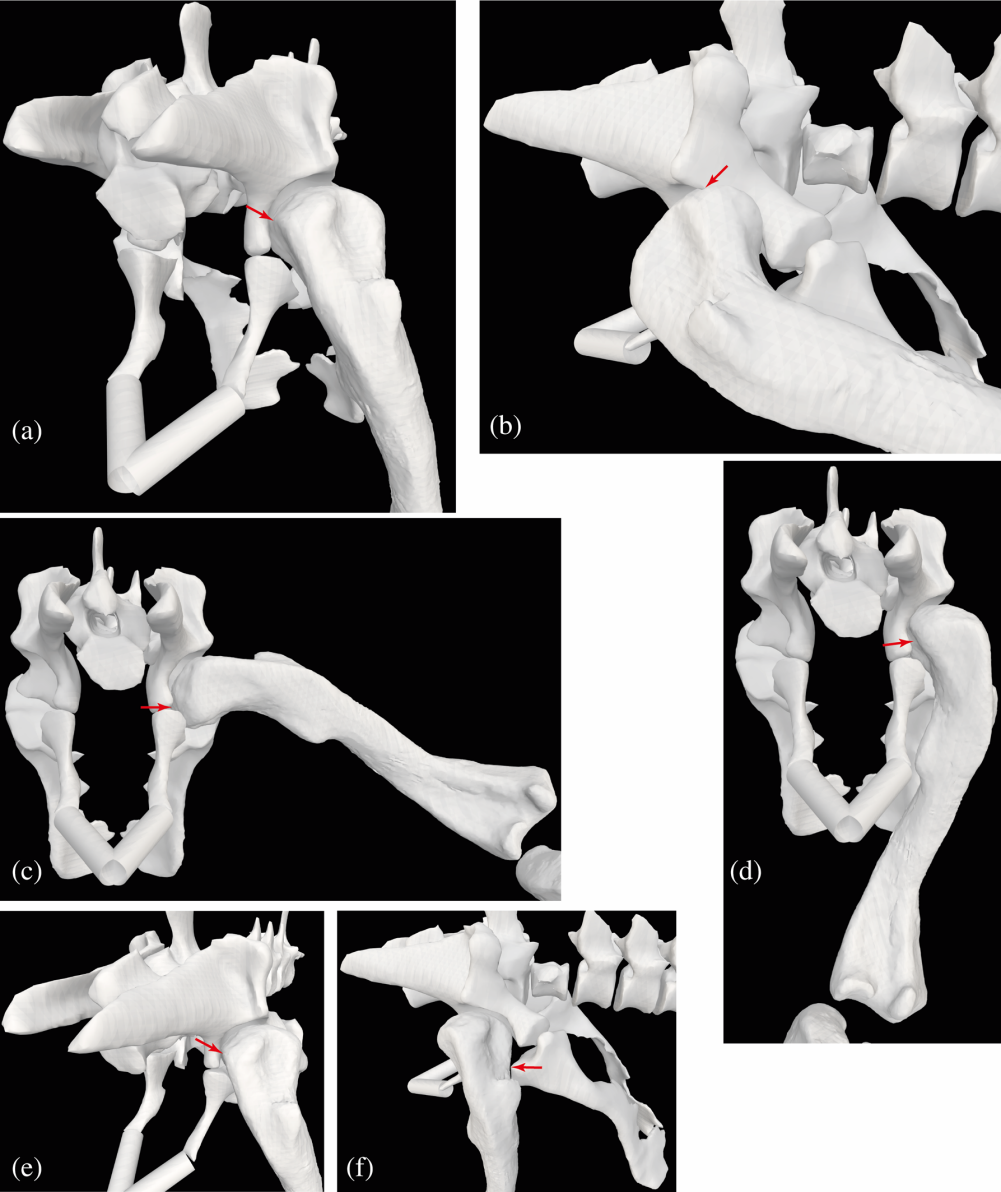
Simple estimates of right hip joint ROMs for *Riojasuchus*; and related morphological traits. Red arrows indicate approximate locations of bone contact or disarticulation used to infer ROM limits. Maximal angles for: (a) hip extension (55°); (b) hip flexion (−65°); (c) hip abduction (−10°); (d) hip adduction (70°); (e) hip external long‐axis rotation (LAR) (50°); and (f) hip internal LAR (−50°).

As Figure 7 shows, *Riojasuchus* lacks the strongly “pillar‐erect” hip joint morphology characterized by Benton and Clark (1988) and Bonaparte (1984) for some Pseudosuchia; involving an acetabulum that faces somewhat ventrally, not simply laterally, and that is deemed to favor a more vertically oriented, strongly adducted femur. Demuth et al. (2020) argued that some amount of pillar‐erect hip function was ancestral for Eucrocopoda (i.e., *Euparkeria*, Archosauria and closely related taxa; Figure 1c), and that remains an accurate description on the basis of a more tightly fitting hip joint than in most earlier Archosauromorpha; but only some suchians such as aetosaurs and ‘rauisuchians’ evolved the most extreme morphology originally described by Benton and Clark (1988) and Bonaparte (1984). There is a modest supra‐acetabular crest, especially craniodorsally, and the femur has a snug fit into the acetabulum, with the main axis of its head oriented craniomedially to medially (PVL 3827/3828: 145/172°; Pintore et al., 2022) versus the mediolateral axis of the femoral condyles. Some prior studies that concluded ornithosuchids had more “semi‐erect” limb postures (e.g., Cruickshank & Benton, 1985; Sullivan, 2015; Walker, 1964) overlooked contradictory traits such as the semi‐perforated, fairly deep acetabulum (see Parrish, 1986) and distinct femoral head, as well as traits of the more distal hindlimb (see below). However, Tsai and Holliday (2014) showed that a perforated acetabulum indicates the presence of pubofemoral and ischiofemoral intracapsular ligaments, rather than an erect hindlimb posture. Future reconstructions of these ligaments could help constrain estimates of hip joint mobility in *Riojasuchus*. Figure 7 depicts the ROMs that we estimated for the hip. To the degree that these are reliable for higher‐level inferences, they suggest relatively wide mobility of the hip.

**FIGURE 8:**
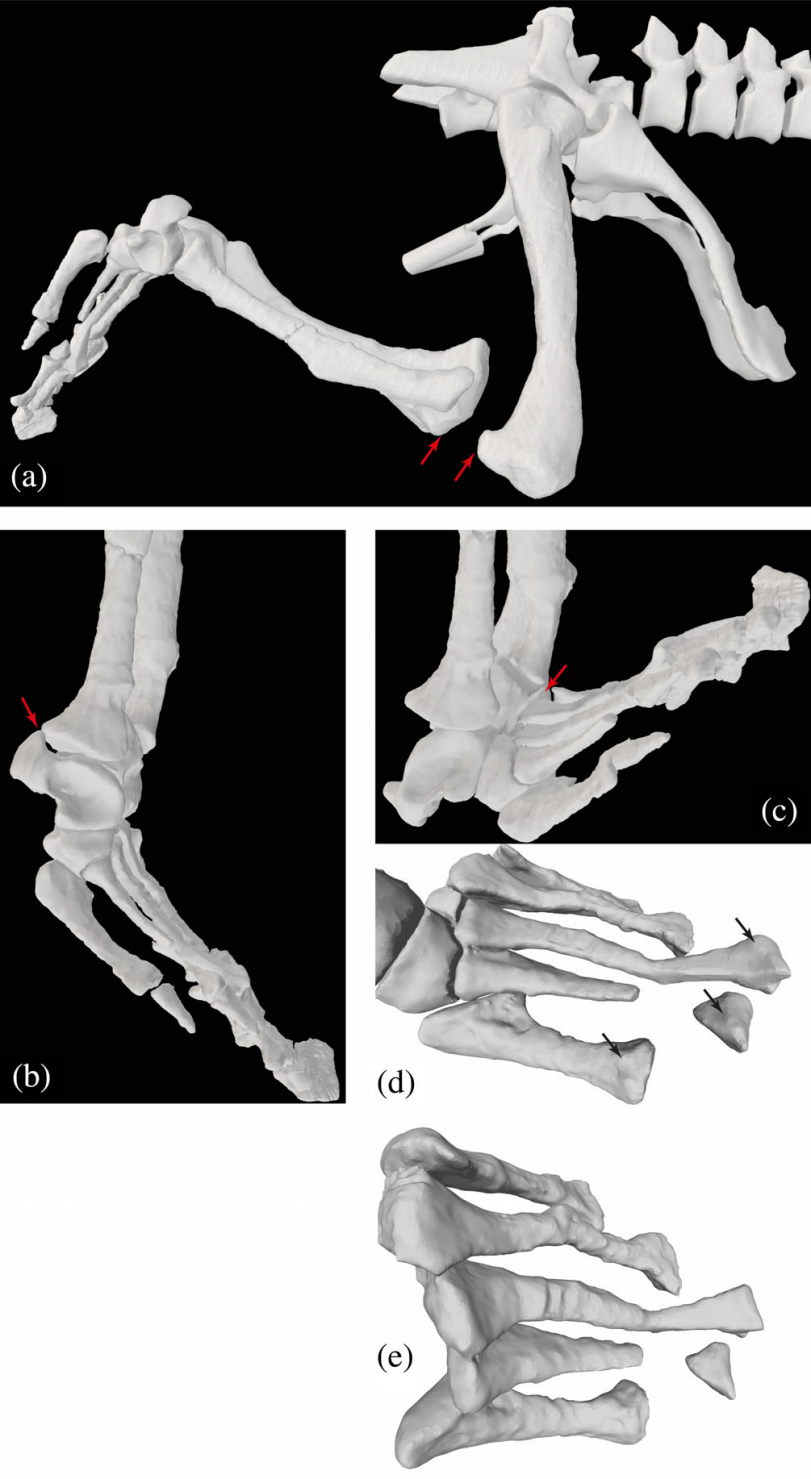
Simple estimates of right knee and ankle joint ROMs, and metatarsal/MTP joint morphology, for *Riojasuchus*. Red arrows indicate approximate locations of bone contact or disarticulation used to infer ROM limits. Maximal angles for: (a) knee flexion (−110°; extension is 0°); (b) ankle extension (30°); and (c) ankle flexion (−50°); then: (d) craniodorsal/lateral oblique view of right pes, showing some “hyperextension” articular surfaces on distal ends of metatarsals (black arrows); (e) proximodorsal view of right metatarsals, showing generally abducted articulation. Not to scale.

The distal end of the femur and proximal tibia have several traits that hint toward more parasagittal knee function. The femur has a deep intercondylar groove and distinct, caudally extensive condyles, and low torsion overall (Parrish, 1986; Pintore et al., 2022). The lateral condyle projects further distally than the medial condyle, which would favor adduction of the tibia and fibula (via a medial incline to the knee axis), thereby facilitating more medial foot placement in a more erect hindlimb posture. There is a prominent, caudally facing fibular condyle (crista tibiofibularis) with a fossa separating it from the lateral condyle on the distal end (Parrish, 1986). However, the morphology of the fibular condyle is not as ornithodiran‐like as Parrish (1986) claimed, and the two specimens (PVL 3827, 3828) strongly differ in the (obtuse) angles of their fibular versus lateral condyles (145/172°) (Pintore et al., 2022). The tibial plateau holds two shallow fossae where menisci would have existed, restricting knee mobility relative to archosaurs with less parasagittal joint function (Parrish, 1986). Figure 8a shows the maximal flexion angle we estimated for the knee.

The distal tibia and proximal tarsals add further evidence for more parasagittal limb (here, ankle) joint function in *Riojasuchus*; such as the tibia positioned vertically relative to the astragalus, with its shaft perpendicular to the mediolateral (flexion/extension) axis of the ankle, and pronounced articular surfaces for the proximal tarsals on the distal fibula, among other traits (see Parrish, 1986). Our estimated joint axes (Figure 3) are less oblique to each other than in *Euparkeria* (Demuth et al., 2020), again more consistent with an erect hindlimb posture and relatively parasagittal gait. Yet the highly apomorphic “crocodile‐reversed” ankle joint of *Riojasuchus* prompts the question of whether its singular morphology might have incurred an unusual ROM or other ankle functions. Parrish (1986) noted its general morphofunctional similarity to other suchian archosaurs with more parasagittal hindlimb function, yet that the more medial orientation of the calcaneal tuber in ornithosuchids might have facilitated a stronger focus on ankle flexion/extension (also Sennikov, 2024). In our model, the calcaneal tuber is at a ~90° angle to the mediolateral axis of the astragalocalcaneal joint, supporting Parrish's point. Yet as in many other plantigrade archosaurs with large calcaneal tubers, the caudal projection of the tuber would have limited ankle extension, because it came into contact with the caudal side of the fibula as the ankle approached hyperextension (Figure 7h). Figure 8b,c shows the ROMs we estimated for the ankle, considered in a comparative context below.

As the metatarsophalangeal joints suffer from some taphonomic distortion and our model only treated them simply here, focussing solely on digit III, we do not address or illustrate their mobility further but made a crude estimate of ROM from −80° plantarflexion to 50° dorsiflexion. However, their morphology might allow for some hyperextension as estimated here—the articular surfaces on the dorsal sides of the distal metatarsals continue well proximally (Figure 8d), so strictly plantigrade digits likely were not maintained (see Clark et al., 1998). The somewhat abducted; not bunched; morphology of the five metatarsals (Figure 8e) indicates a typically plantigrade pes—the full proximal halves of the metatarsals (particularly II–IV) are not in contact as they are in digitigrade ornithodirans and ‘sphenosuchian’ crocodylomorphs (Turner & Gatesy, 2021). The fifth metatarsal remains relatively long, but the proximal hook incurring more lateral splay is absent. That morphology is consistent with a plantigrade pes but a more parasagittal function of the autopodium (e.g., Parrish, 1986; Sennikov, 2024; Sereno, 1991). Nonetheless, 3D motions within the tarsals and metatarsals were likely (see Brinkman, 1980; Turner & Gatesy, 2021, 2023) but not modeled here. Parrish (1986) pointed out some features of the intratarsal joints that suggested more parasagittal function distal to the ankle, as well.

Overall, our qualitative and quantitative inferences drawn from our 3D model of *Riojasuchus* (Figures 7 and 8), while deserving more comprehensive analyses, favor the interpretations that it used a relatively erect hindlimb posture and more parasagittal gait, but with a plantigrade pes. Comparisons of our estimates for ROMs in *Riojasuchus* to studies of other non‐avian archosaurs reinforce this conclusion. For example, ROMs of the Triassic theropod dinosaur *Coelophysis bauri* are somewhat similar, with the hip having −83° flexion to 60° extension, −21° adduction and 62° abduction, 60° internal and −37° external rotation; the knee 0° to −144° flexion; the ankle (values adjusted to our model's convention of pes with 90° angle to crus as plantigrade = 0°) 90° extension to −29° flexion; and the third MTP joint 17° dorsiflexion to 180° plantarflexion (Bishop, Cuff, & Hutchinson, 2021). Of these estimated ROMs, only the ankle and MTP joint stand out as remarkably different, although it is somewhat surprising that hip ROMs are rather similar despite the conspicuously more substantial supraacetabular crest in *Coelophysis*. The ankle had a greater capacity for flexion but much less for extension in *Riojasuchus*. The former is because of the long, broad “roller” surface on the proximal calcaneus for articulation with the distal fibula (and a fossa on the craniodistal astragalus that receives the proximal metatarsals; “astragalar hollow” of Cruickshank, 1979; “central concavity” of von Baczko et al., 2020) in *Riojasuchus* (Figure 8c; see also Cruickshank & Benton, 1985; Parrish, 1986; Turner & Gatesy, 2023). The latter, as noted above, is explained by the large calcaneal tuber in *Riojasuchus*, whose absence facilitates the digitigrade pes in Dinosauriformes, although this issue remains to be well‐explored in digitigrade suchians such as some poposauroids and ‘sphenosuchians’. The very different MTP ROMs appear to be caused by the judgment that the third metatarsal's distal articular surface is more extensive dorsally/cranially in *Riojasuchus* versus *Coelophysis* but less extensive ventrally/caudally (e.g., Figure 8d,e), although we admit that this judgment is highly subjective and constrained by the low mesh resolution of the distal metatarsals.

Manafzadeh et al. (2021) provided valuable maximal hip joint mobility data for *Alligator mississippiensis*: for the hip, about −100/100° flexion/extension, 5/90° adduction/abduction and −180/180° internal/external rotation; thus our ROM estimates for *Riojasuchus* are more restricted in flexion/extension and long‐axis rotation, as the morphology suggests. Brinkman (1980) manipulated cadaveric feet of *Caiman sclerops*, measuring about −65 to 25° flexion/extension ROMs of the ankle (astragalocalcaneal joint). This is a similar total ROM (90° vs. 80° here) but with slightly greater flexion ROM in *Caiman*, possibly due to the absence of soft tissues in our model's joints. Demuth et al.'s (2020) maximal joint mobility estimates for the hip of *E. capensis* were about −90/90° flexion/extension, 0/125° adduction/abduction and −10/40° internal/external rotation; so the hip of *Euparkeria* seems to have been more mobile than in *Riojasuchus* except in long‐axis rotation. More conclusive inferences about comparative and evolutionary joint mobility will require more cutting‐edge methods (e.g., Bishop et al., 2023; Demuth et al., 2020; Manafzadeh et al., 2024) but could easily use our model's geometry.

While we do not focus here on modeling forelimb function in *Riojasuchus*, our model could be adapted to do so in more detail in the future. Regardless, understanding forelimb function is vital for inferring whether bipedal locomotion was obligate, facultative, or impossible. The well‐preserved forelimbs of *Riojasuchus* (Figures 1 and 2) give qualitative clues in this regard. Sereno (1991) noted the slender morphology of the radius and ulna relative to the metacarpals, and slightly elongate ulnare and radiale that are reminiscent of those of crocodylomorphs. The high ‘quadrupedality index’ (i.e., similar forelimb vs. hindlimb lengths, relative to bipedal dinosaurs) calculated for *Riojasuchus* by Kubo and Kubo (2012), along with the elongate proximal carpals, suggests quadrupedalism, and perhaps the gracile forearm relates to more weight being carried by the hindlimbs (rather than bipedalism itself), as the postcranial morphology and our COM estimate suggest. However, the carpal region of *Riojasuchus* is markedly different from that of Crocodylia—unlike in the latter, the radius and ulna, and radiale and ulnare, were of roughly equal functional lengths (i.e., not a longer ulna and radiale). Thus *Riojasuchus* lacked an asymmetrical antebrachiocarpal joint that facilitates manus abduction during stance phase in Crocodylia, and more parasagittal forelimb function as well as a distinct automatic wrist folding mechanism (see Hutson & Hutson, 2014; Pashchenko, 2022). Furthermore, digit I is much more robust than the other digits, with II–V progressively smaller. If ornithosuchids did use quadrupedalism they may simply have used more erect forelimbs rather than the complex mechanism in Crocodylia. This putative erect forelimb posture in *Riojasuchus* also is evidenced by traits such as the caudally facing glenoid (Figure 2i).

### 3.3 Do fossil trackways of ornithosuchids exist?

It would be valuable to relate our modeling results and inferences about foot/limb orientations to ichnological data derived from fossil trackways assigned to ornithosuchids, but as often is the case, such assignments are ambiguous. Lack of preservation of many manual and pedal phalanges in known ornithosuchid fossils worsens the ambiguity. Older studies assigned some *Chirotherium* (e.g., *C. lulli*) trackways to “ornithosuchids” (e.g., Baird, 1954, 1957; Swinton, 1961), but this was an ambiguous phylogenetic context that also included taxa such as *Euparkeria* (now known as a non‐archosaurian archosauriform; e.g., Gauthier, 1986; Sereno, 1991 and subsequent studies), and that ichnogenus was and remains thought to potentially pertain to numerous archosaur clades. More recently, studies such as Gand et al. (2007) and Klein and Lucas (2021) considered some tracks such as *Sphingopus ferox* as potentially made by “ornithosuchids” (again with some ambiguity of meaning), even moving bipedally; and as Padian et al. (2010) noted, Haubold (1986) posited that *Parachirotherium* trackways were made by ornithosuchids; possibly as bipeds. The tridactyl pes retaining digits I and V with some splay of both, and digit III as longest, are features that might match numerous kinds of trackways, although to our knowledge none exhibit the distinctly blade‐like pedal unguals—and show whether the manus contacted the ground, which would be a vital independent test of bipedalism. Further detailed, synapomorphy‐based analyses of ichnological data are needed to convincingly assign any trackways to ornithosuchids, in our view, and such a finding would be of great relevance to our study.

### 3.4 Hindlimb myology

As Walker (1964, 1977) noted, the lesser trochanter of ornithosuchids is an unusual structure important for inferences about hip joint, thigh muscle and limb functional morphology—and their evolution. A key question about this structure has long been what muscle it is an osteological correlate of. As per Romer (1923b) and many subsequent studies (see Hutchinson, 2001a), we follow the inference that M. iliofemoralis (IF) inserted on the lesser trochanter, presumably in any archosaurs having it. This invokes the assumption that the IF insertion moved proximally and cranially on the femur in archosaurs that evolved a lesser trochanter. The proximodistal striations typically seen on lesser trochanters are consistent with the insertion of a muscle originating from the lateral ilium dorsal to the acetabulum, and these are evident in *Riojasuchus*. Those striations are not consistent with the alternative hypothesis, that PIFI2 (which already has a proximal insertion in Reptilia) inserted there, as the PIFI2's origin was craniomedial (or cranial, if the PIFI2's origin was presumed to be preacetabular and lateral) to the lesser trochanter. Unlike in Dinosauriformes/Avemetatarsalia, the lesser trochanter of Ornithosuchidae (and Crocodylomorpha; convergently) is not associated with a trochanteric shelf, inferred as an osteological correlate of the split of the ancestrally single IF into two muscles (M. iliotrochantericus caudalis and M. iliofemoralis externus), and constraining the location of the ISTR's insertion on the proximolateral femur (Hutchinson, 2001a; Hutchinson & Gatesy, 2000; Nesbitt et al., 2017). Thus we do not infer a split of the IF muscle into two parts in *Riojasuchus*. Otherwise, we did not notice any exceptional muscle scars or other osteological correlates on the *Riojasuchus* specimens.

Our reconstructed hindlimb musculature of *Riojasuchus* (Figures 4 and 5) provides a reminder that muscle moment arms are not enough to quantify moment‐generating capacities around limb joints, although they are valuable for estimating muscle actions. The attachment sizes (especially origins) of muscles on bones presumably provide at least qualitative clues to the sizes and thus force‐generating capacities of those muscles (see Cuff et al., 2023). Most notably in *Riojasuchus*, the broad, deep pubic ‘aprons’ provided ample space for the PIFE1 + 2 origins (e.g., Figure 4b), suggesting relative expansion of those muscles' sizes (see also Parrish, 1986) and thus moment‐generating capacities. The ischium, even though incomplete, was clearly not as extensive and thus some muscles such as ISTR, ADD1 + 2, and PIFE3 likely were not so expanded. Otherwise, many hindlimb muscles do not seem to have been unusually large for an early archosaur, as far as pelvic/long bone shapes suggest.

Next, we compare our reconstruction of hindlimb musculature in *Riojasuchus* (Table 2; Figures 4 and 5) with those for two other suchians (as follows), and the archosauriform *Euparkeria*, that have used similar methods (i.e., the EPB with osteological correlates). It is no surprise that these published reconstructions have broad similarities with ours. Non‐avian (including some dinosaurian) hindlimb myology seems to have been fairly conservative, and methods and necessary evidence are well established, so results should be similar except where there are drastic differences in morphology or assumptions. Differences from *Euparkeria* (Demuth et al., 2022) largely are due to more plesiomorphic traits in the latter (e.g., lack of a lesser trochanter; pelvic morphology); the two studies fundamentally used the same dataset.

We agree with Liparini and Schultz (2013), who studied the “rauisuchian” *Prestosuchus chiniquensis*, that the origin of the PIFI1 is somewhat ambiguous in early archosaurs, but we infer a more plesiomorphic origin on the medial ilium for *Riojasuchus*, as there is no preacetabular “cuppedicus” fossa (e.g., as in some theropods) suggesting a derived, lateral shift (Hutchinson, 2001b). Here, as per Liparini and Schultz (2013), we disagree with the PIFI1 reconstruction by Schachner et al. (2011) and Bates and Schachner (2012) for *Poposaurus gracilis*; it remains unclear if the PIFI1's origin shifted laterally (and solely onto the ilium) in any non‐theropods, in our view. Similarly, following Hutchinson (2001b) and later studies, we discern no evidence for a lateral shift of the PIFI2 origin onto the ilium (again, as in most or all non‐theropods). An origin from the vertebral region closest to the pelvis is most parsimonious, as a broad space remains open beneath the ventral edge of the preacetabular ilium, through which the PIFI2 path crosses in Crocodylia (whereas in Aves it has become ‘captured’ by the preacetabular ilium closing off that space; Hutchinson, 2001b). Walker (1977) reconstructed some thigh musculature in *Ornithosuchus*, using a less explicit method than the EPB. His reconstruction is very similar to ours except that it placed a PIFI “ventralis” (using a lepidosaurian than archosaurian homologue) where we (like Romer, 1923b and most other studies) infer the PIFE1 to have originated, from the cranial surface of the pubic apron.

Bates and Schachner (2012) placed the PIFE1–3 origins differently from ours—mostly more distally, contrary to other recent reconstructions in archosaurs (e.g., Allen et al., 2021; Carrano & Hutchinson, 2002; Hutchinson, 2001a, 2002). The IF insertion reconstructed in *Prestosuchus* is more craniad on the femur than inferred in other archosaurs (e.g., Schachner et al., 2011), in which it is more caudad (e.g., in Crocodylia, with corresponding osteological correlates in some non‐ornithodirans; Hutchinson, 2001a) or else shifted proximally onto a lesser trochanter, as we reconstruct for *Riojasuchus* like (convergently) in dinosauriforms (Hutchinson, 2001a) as well as (apparently) ‘sphenosuchian’ crocodylomorphs. Unlike Liparini and Schultz (2013), we judge that there is no strong case for two heads of the AMB as this appears to be an apomorphy of Crocodylia and some ratites (see Bishop, Cuff, & Hutchinson, 2021; Hutchinson, 2002). Liparini and Schultz (2013) placed the ISTR insertion more medially on the femur than in extant archosaurs or evidence from fossil archosaur femora (Hutchinson, 2001b).

The “craniomedial fossa” for the PIT and FTI1 insertion on the tibia noted by Liparini and Schultz (2013) is unusually distal and cranial for archosaurs (e.g., see Cong et al., 1998 for *Alligator sinensis*, which our dissections of Crocodylia agree with; also Schachner et al., 2011), so we are uncertain of what soft tissue it might correlate with. Scars more similar to our reconstructed insertions of the PIT and FTI1 are present in numerous other archosauromorph specimens (e.g., the early archosauromorph *Azendohsaurus*, Nesbitt et al., 2015; the pseudosuchian *Nundasuchus*, Nesbitt et al., 2014; and the aphanosaur *Teleocrater*, Nesbitt et al., 2017).

For further discussion of detailed differences between our dataset and the inferences of Liparini and Schultz (2013), see the supplementary material in Bishop, Cuff, and Hutchinson (2021). Notably however, as Wilhite (2023) has shown in great detail, the CFL should have originated from the transverse processes and especially chevrons of the more proximal caudal vertebrae, not from the centra as many prior studies have reconstructed it. Here, as in most other studies of fossil archosaurs, we do not reconstruct the secondary tendons (to the lower limb) of the CFL and some other muscles (e.g., AMB, FTE), as their functional importance remains unclear and some of them can be considered instead as tendons of origin of lower limb muscles (e.g., GE) that are reconstructed here. Distal hindlimb muscles reconstructed by Schachner et al. (2011) agree well with ours. Other differences between studies are minor issues relating to subjective interpretations of topological positions (e.g., of broad muscle attachments or those lacking distinct osteological correlates). Overall, we expect that the mostly minor disagreements noted here would generally result in modest deviations of functional interpretations.

### 3.5 Hindlimb muscle moment arms and the evolution of archosaurian muscle actions

Because *Riojasuchus* had an odd mixture of plesiomorphic archosaurian, derived suchian and highly apomorphic ornithosuchid morphological traits, estimating its MMAs should aid insights into some basic functional consequences of those traits. These MMA data are valuable not only in the context of this taxon's palaeobiology (e.g., controversies about posture) but also in comparative (e.g., with other archosaurs having similar derived traits) and evolutionary (e.g., locomotor adaptations in pseudosuchians vs. ornithodirans; and the dinosaurian “locomotor superiority hypothesis”; see Cuff et al., 2022) contexts. Here we first consider the MMA outputs of our model including their dependencies on joint orientations, then review how any remarkable morphological traits relate to these data, and then compare these results to those for other archosauriforms.

**FIGURE 9:**
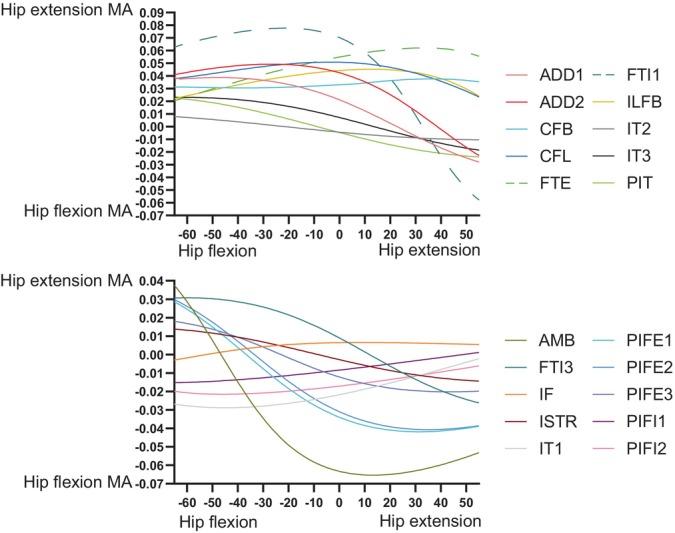
MMAs for hip flexion and extension in the *Riojasuchus* model, showing variation with hip flexion/extension angle.

**FIGURE 10:**
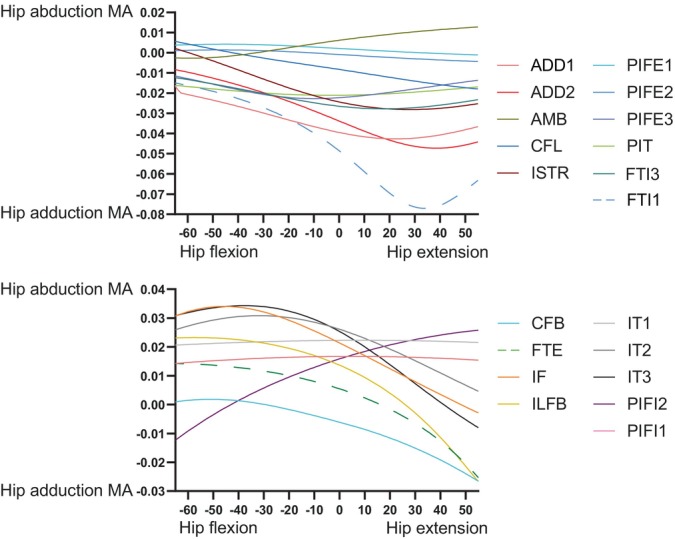
MMAs for hip adduction and abduction in the *Riojasuchus* model, showing variation with hip flexion/extension angle.

**FIGURE 11:**
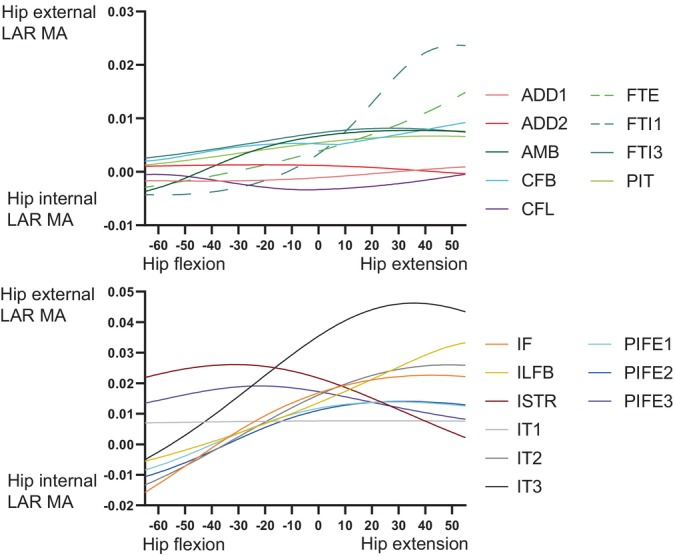
MMAs for hip internal and external long‐axis rotation (LAR) in the *Riojasuchus* model, showing variation with hip flexion/extension angle.

Figure 9 depicts hip flexor and extensor MMAs, showing that the major flexors were AMB, IT1, and PIFE1 + 2; whereas the major extensors were FTI1, FTE, CFL, and ILFB. Many muscles, however, became hip flexors with increasing hip extension, although mostly near extreme extension. Indeed, MMAs tended to decrease (become weaker in hip extension/stronger in flexion) with increasing hip extension. FTI1, ADD1 + 2 and FTI3 were the major hip adductors, versus IT1–3 and IF as the major abductors; and most muscles switched from abduction to adduction (or at weaker abduction) with increasing hip extension, except AMB and PIFI2 (Figure 10). There were surprisingly few muscles with MMAs consistently for much internal LAR of the hip (see also Allen et al., 2021); ADD1, CFL, and IT1 had modest but consistent MMAs for internal LAR (Figure 11). IT3, ISTR, and FTI1 (with an extended hip joint) were among the strongest external rotators of the hip. Again, numerous muscles switched actions; often with internal LAR becoming external LAR with increasing hip extension.

**FIGURE 12:**
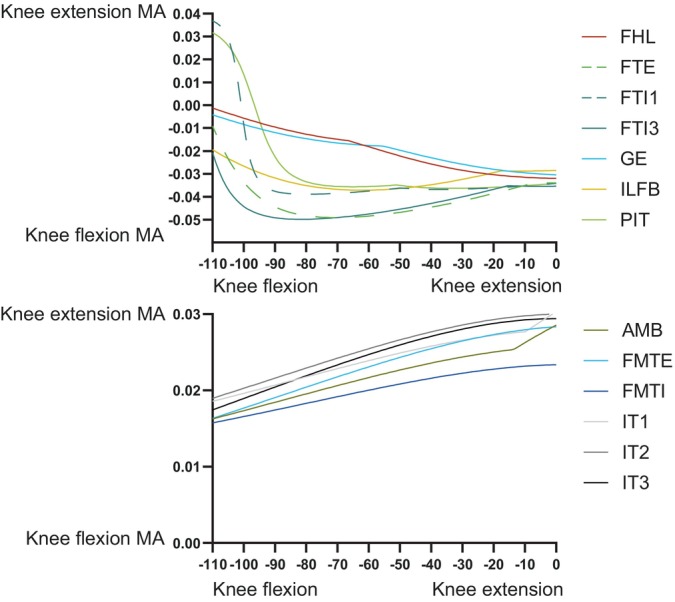
MMAs for knee flexion and extension in the *Riojasuchus* model, showing variation with knee flexion/extension angle.

**FIGURE 13:**
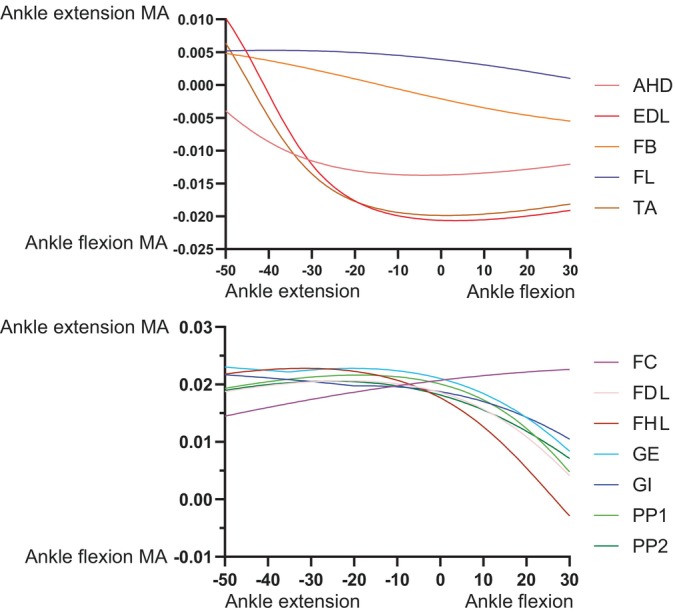
MMAs for ankle flexion and extension in the *Riojasuchus* model, showing variation with ankle flexion/extension angle.

**FIGURE 14:**
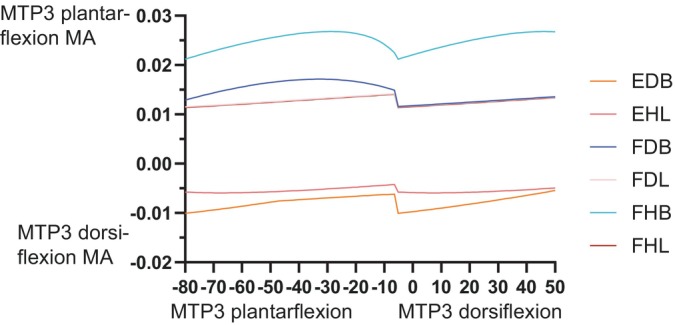
MMAs for MTP3 joint plantarflexion and dorsiflexion in the *Riojasuchus* model, showing variation with MTP3 flexion/extension angle. FDL's and FHL's curves are overlapping.

At the knee, trends were fairly simple (Figure 12). The typical knee flexors of the “hamstrings” muscle group remained strong (FTE and FTI3 most prominently) in that DOF (flexor MMAs increasing with knee extension; with FTI1 and PIT switching actions), whereas the “triceps femoris” (IT1–3, AMB, FMTE, and FMTI) were antagonistic to the “hamstrings”, their knee extension MMAs becoming greater with knee extension. Likewise, ankle MMAs (Figure 13) followed basic functional groupings. The major ankle extensors were FC, GE, and FHL, but essentially all anatomical ankle extensors had similar capacities, which tended to decline with extreme ankle flexion. Only the EDL, TA, and AHD were ankle flexors, exhibiting MMA versus joint angle trends opposite to those of the extensors. The MTP3 joint's muscles (Figure 14) revealed some weaknesses of our simple modeling of the pedal digits. Whereas the EDB and EHL were clear dorsiflexors and FHL, FDL, FDB, and FHB remained plantarflexors, their MMA patterns were not smooth, caused by the simple modeling of wrapping surfaces and via points of muscle paths around the MTP3 joint. More complex models of the pedal digits would resolve these issues, although as the intrinsic pedal muscles (all but FDL and FHL) would not be expected to be large in *Riojasuchus* (they are relatively small in extant Crocodylia; e.g., Brinkman, 1980), the functional consequences of some amendments might not be substantial.

Our MMA analyses (Figures 9, 10, 11, 12, 13, 14) reinforced the functional importance of key morphological traits of the hindlimbs of *Riojasuchus*. Most importantly, as per Section 3.4, the lesser trochanter's role as the insertion for the IF underlies that muscle's consistent action in hip abduction as well as some capacity for extension and external LAR.

Cuff et al. (2022) provided a useful dataset for early archosauriform (and extant crocodylian) hindlimb MMAs from new models and previous studies. Three species of Crocodylia (*A. mississippiensis*, *C. johnstoni*, and *C. niloticus*) have available models (Allen et al., 2021; Bates et al., 2015; Bates & Schachner, 2012; Cuff et al., 2022; Wiseman et al., 2021). Furthermore, there are models of the extinct archosauriforms *Euparkeria* (Demuth et al., 2022; Demuth, Wiseman, & Hutchinson, 2023), *Batrachotomus* (Cuff et al., 2022), *Poposaurus* (Bates et al., 2015; Bates & Schachner, 2012; Cuff et al., 2022), *Marasuchus*/*Lagosuchus* (Allen et al., 2021; Cuff et al., 2022), and *Coelophysis* (Allen et al., 2021; Bishop, Cuff, & Hutchinson, 2021; Cuff et al., 2022). See those studies for details. Most differences will pertain to the proportions of the pelvis (e.g., cranial expansion of the preacetabular ilium, or lack thereof; Carrano, 2000) or subjective modeling assumptions. Yet as Cuff et al. (2022) showed, further archosaurian musculoskeletal specializations can be revealed with a comparative approach, and as per Allen et al. (2021), these can be placed into a phylogenetic context. Following Cuff et al. (2022), if *Riojasuchus* was bipedal (as in *Poposaurus*) we would expect (vs. quadrupedal *Batrachotomus*) larger ankle extensor MMAs as well as generally larger hip flexor/extensor MMAs. Cuff et al.'s (2022) non‐dimensionalised MMA data are distinctly greater for *Poposaurus* than our results for *Riojasuchus* are: 77%, 17%, and 36% greater for hip flexors (PIFI1 + 2), hip extensors and major ankle extensors. These findings certainly are consistent with the much more expansive pelvis of *Poposaurus* (explaining the small PIFI1 hip flexor moment arm in our *Riojasuchus* model), and would support an inference that *Riojasuchus*'s hindlimb MMAs were not well specialized for bipedalism and/or digitigrady as in *Poposaurus*. Overall, among archosauriform models currently available, the non‐dimensionalised MMAs of the hindlimb of *Riojasuchus* compare best with those of *Batrachotomus*.

Table 3 compares general patterns for which muscles tend to have the greatest MMAs across the early Archosauriformes studied. Several more patterns emerge: the same muscles tend, with a few exceptions that might pertain more to modeling assumptions than to morphological differences, have the largest MMAs for each DOF. This tendency applies to the hip flexors (IT1, AMB, and PIFI2; PIFE1 + 2 sometimes), hip extensors (FTI1, FTE, and CFL/CFB; ADD1 + 2 sometimes), hip adductors (ADD1 + 2, FTI1), hip abductors (IT1–3, IF/IFE, and PIFI2), hip internal rotators (variable, but often PIFE1 + 2, PIFI1 + 2, and FTI1/2) and hip external rotators (ISTR, PIFE3, and IT3). These patterns broadly agree with prior studies using qualitative functional morphology (e.g., Hutchinson & Gatesy, 2000; Walker, 1977). Yet while typical muscle actions are conservative among early archosauriforms (and retained in Crocodylia), magnitudes of MMAs relative to body size clearly evolved across the clade, and in some cases the qualitative actions evolved (see Allen et al., 2021; Bates et al., 2015; Bates & Schachner, 2012; Cuff et al., 2022), although we did not detect obvious derived MMAs that might be related to bipedalism in *Riojasuchus*.

**TABLE 3:** Comparison of largest MMAs for key hip muscles in 3D musculoskeletal models of early archosauriforms' hindlimbs published to date.

Taxon	Major MMAs					
	Hip flexors	Hip extensors	Hip adductors	Hip abductors	Hip internal LAR	Hip external LAR
*Riojasuchus*	AMB, IT1, PIFE1 + 2	FTI1, FTE, CFL, ILFB	FTI1, ADD1 + 2, FTI3	IT1–3, IF	ADD1, CFL, IT1	IT3, ISTR, FTI1
*Euparkeria*	IT1, AMB, PIFI1	FTI1, FTE, IT3	ADD1, ADD2, PIT	IT2, IT3, ILFB	FTI2, FTI1, PIFI2	PIFE3, ISTR, IT3
*Batrachotomus*	AMB, IT1, PIFI2	FTI1, CFL, ADD2	FTI1, ADD2, ADD1	IT2, IF, PIFI2	PIFE2, PIFE1, FTI1	PIFE3, ISTR, CFL
*Poposaurus*	AMB, IT1, PIFI2	CFL, FTE, FTI1	FTI1, ADD1, ADD2	IT1, IF, IT2	PIFI2, ADD1	FTE, IT3, PIFE3
*Crocodylus*	IT1, AMB, PIFI1	CFL, CFB, FTI1	FTI1, ADD1, ADD2	IT3, IF, FTE	PIFI2, AMB, FTI2	IT3, ISTR, PIFE3
*“Marasuchus”*	PIFE1, PIFE2, AMB	FTI1, CFL, ADD1	ADD1, FTI1, ADD2	IT3, IFE, IT2	IT1, PIFI2, FTI1	ISTR, PIFE3, PIFE2
*Lesothosaurus*	IT1, AMB, IT2	FTE, CFL, ADD1	ADD1, ADD2, PIFE1	IT2, IFE, IT3	PIFI2, ITC, FTE	ISTR, PIFE3, PIFE2
*Plateosaurus*	AMB, IT1, PIFE2	FTE, CFB, CFL	FTI1, ADD2, ADD1	IT2, IT3, IFE	PIFI2, PIFE1, FTE	ISTR, CFL, PIFE3
*Coelophysis*	IT1, AMB, PIFI2	FTI1, ADD2, FTE	FTI1, ADD2, ADD1	IT3, IT2, ILFB	PIFI2, ITC, PIFI1	ISTR, PIFE3, FTI1

*Note*: Approximately ordered from largest to smaller magnitudes. See main text for references; and Table 2 for muscle acronyms. “*Marasuchus*” ~ *Lagosuchus* by some studies.

Intriguingly, while *Riojasuchus* has a lesser trochanter; as did the first bipedal Dinosauriformes; and the bipedal *Poposaurus* does not, nor does the apparently quadrupedal *Batrachotomus*, the IF muscle tends to switch from an internal to an external rotator of the hip with increasing hip extension in models of all of them; that is, its action remains similarly dependent on hip orientation. However, as the IF muscle splits into its two “avian” parts in Dinosauriformes, models suggest that the most cranial components (of M. iliotrochantericus caudalis) tend to remain internal rotators at most or all hip angles (data from Allen et al., 2021; Cuff et al., 2022). Thus a split of the IF into two muscles combined with preacetabular expansion (and perhaps changes of the proximal femur) seems to have favored transformation of the IF components into having enhanced internal rotator capacities, as they do in extant Aves (see Allen et al., 2021 for further discussion). The key functional shift with the origin of bipedalism, then, appears to have involved altered timing of neural excitation of the IF components (Hutchinson & Gatesy, 2000) more than altered MMAs, although there are hints of some morphofunctional transformations of the IF muscle complex (Allen et al., 2021). Presence of a lesser trochanter in fossil archosaurs thus is not in itself indicative of bipedalism.

## 4 CONCLUSIONS

Here, we have reconstructed one of the few currently existing musculoskeletal models (or even hindlimb myology) of a pseudosuchian archosaur. First, we used this model to address our major aims about potential hindlimb orientation (e.g., erect posture), joint functions, and bipedalism. Our findings support what appears to be a relative consensus today that ornithosuchids such as *Riojasuchus* used a roughly erect (adducted) posture and parasagittal gait, with a plantigrade pes. However, as per prior studies we found conflicting or ambiguous evidence about potential bipedalism in *Riojasuchus*—some aspects of limb and body dimensions (including the COM) are consistent with (but not fully indicative of) bipedalism, whereas others such as forelimb morphology are not. The three sacral vertebrae of *Riojasuchus* might be added to evidence for bipedalism, as such a trait mainly is found in bipedal archosaurs, but there is as of yet no mechanistic justification for why three sacral vertebrae indicate bipedalism. Nonetheless, we have not addressed axial musculature in our model, and the tall, robust neural spines of the dorsal vertebrae in *Riojasuchus* (see von Baczko et al., 2020) might have helped support the body against nose‐down pitching moments during bipedalism (e.g., Christian & Preuschoft, 1996; Demuth, Wiseman, & Hutchinson, 2023), yet testing this possibility would require more sophisticated modeling. There is a need for consilience from multiple independent lines of evidence: unfortunately, unambiguous ichnological data from ornithosuchids is lacking, but analyses of forelimb function could be helpful, although we suggest how forelimb morphology in *Riojasuchus* at least indicates an erect posture. Our model does not show any remarkable function of the “crocodile‐reversed” proximal tarsal joint versus a “crocodile‐normal” one, but does reveal functional differences between these two morphologies and the “advanced mesotarsal” morphology of Avemetatarsalia (e.g., lesser ankle mobility for extension but greater for flexion).

Second, we reconstructed the hindlimb myology of *Riojasuchus* for usage in estimating the MMAs of the major muscles and their functional dependence on joint orientations. In comparing our findings to those for other archosauriforms, we noted a general agreement of inferences about myology and MMA patterns; suggesting good repeatability of these methods but also conservatism in the form and function of hindlimb muscles in many non‐avian archosaurs (also see Bates et al., 2015). It is noteworthy, though, that the MMAs we obtain are relatively smaller than in *Poposaurus* but similar to *Batrachotomus*, which might be additional evidence for quadrupedalism in *Riojasuchus*. Importantly, we argue that the presence/absence of a lesser trochanter on the femur for the IF muscle insertion, while consistent with an abductor action, is not sufficient evidence for bipedalism in archosaurs.

Most importantly, our study identifies the need for more quantitative functional analyses of locomotor function in *Riojasuchus* to settle some of the mysteries noted above (especially bipedalism), to delve deeper into estimating muscle functions, and to ask bolder questions such as about maximal locomotor performance, stability or efficiency, and their tradeoffs with morphology versus those in other early Mesozoic taxa. Predictive simulations (e.g., Bishop, Falisse, et al., 2021; see Demuth, Herbst, et al., 2023) are an example of the more cutting edge approaches that could satisfy this need to deliver insights. Such insights could even help resolve major macroevolutionary controversies, most prominently the causal factors underlying extinction versus survival of archosaurian lineages across the Triassic–Jurassic boundary (i.e., the “locomotor superiority hypothesis” reviewed by Cuff et al., 2022). If ornithosuchids had some capacity for bipedalism, and if bipedalism was an advantage for early dinosaurs, why did they and some other suchian archosaurs go extinct? While it is presumed that such taxa were “failed” experiments in bipedalism, this still begs the question why they “failed”, which evolutionary biomechanical analyses can help address. However, models and simulations are not enough. Fundamental morphological data (e.g., osteological correlates), qualitative functional analyses (to constrain and inform quantitative ones), fossil trackway data and other information all remain vital for achieving a strong consensus, and our study has moved toward attaining that goal.

## AUTHOR CONTRIBUTIONS

**M. Belen von Baczko:** Funding acquisition; investigation; writing – review and editing; data curation; resources; visualization. **Juned Zariwala:** Investigation; funding acquisition; writing – review and editing. **Sarah Elizabeth Ballentine:** Investigation; writing – review and editing. **Julia B. Desojo:** Funding acquisition; writing – review and editing; supervision. **John R. Hutchinson:** Conceptualization; investigation; funding acquisition; writing – original draft; methodology; validation; visualization; writing – review and editing; software; formal analysis; project administration; data curation; supervision; resources.

## Supporting information


**APPENDIX S1:** Supporting information.

## Data Availability

CT data of the postcranial elements are available at MorphoSource.org (https://www.morphosource.org/projects/000618625?locale=en). The OpenSim musculoskeletal model is available at Figshare (https://figshare.com/s/40dcd37cae743618314d; doi: 10.6084/m9.figshare.25921495).
